# Supplementary material for: Cost-effectiveness of decellularised bone allograft compared with fresh-frozen bone allograft for acetabular impaction bone grafting during a revision hip arthroplasty in the UK
Source: BMJ Open. 2023 Oct 6;13(10):e067876. doi: 10.1136/bmjopen-2022-067876 (PMC10565200; doi:10.1136/bmjopen-2022-067876)
Supplement: Supplementary data [file bmjopen-2022-067876supp001.pdf]

Supplementary Table 1: Cost of treatments: all costs are in £ (GBP) and converted into 2022 prices [1]

		Fresh-frozen allograft	Decellularised allograft	Source
Pre surgery	Inpatient stay cost (£)	6929.31		[2]
	Investigation costs	644.58		[3]
	Drug costs	490.84		[3]
Surgery	Implant costs	3593.18		[3]
	Theatre costs	1711.66		[3]
	Inpatient costs	235.44		[4]
	Impaction materials	617.50		[5]
	Total graft costs (2.43 femoral head grafts)	1724.59 [a]	24398.95 [b]	[a] [6]<br>[b] Table 1
Post-surgery costs	Out-patient costs	340.21		[4]
	Aids and adaptations	25.89		[4]
	Medication	29.58		[4]
**Total cost**		**16342.79**	**39017.15**	

Supplementary Table 2: Costs for each reagent used during decellularisation manufacturing: calculated from current suppliers. All costs are in £ (GBP) and converted into 2022 prices [1]

Reagent	Cost per graft
Aprotinin	85.34
Benzonase	238.69
Amphotericin B	6.57
Penicillin-Streptomycin	47.21
Phosphate-buffered saline	91.46
Nuclease Buffer	31.82
Hypertonic Buffer	31.82
Detergent Buffer	29.28
Hypotonic Buffer	29.28
**Total**	**591.47**

Supplementary Table 3: Transition Probabilities: Transition probabilities for all health states for both bone graft choices, *Dependent upon time period of model (year 1 to year 28 shown)

Fresh-frozen allograft				
	To			
**From**	**1st revision**	**Post-revision**	**Re-revision**	**Death**
**1st revision**	0.0000	0.668 – 0.927*	0.026	0.0466 – 0.306*
**Post-revision**	0.0000	0.061 – 0.954*	0.026	0.0195 – 0.364*
**Re-revision**	0.0000	0.6675 – 0.927*	0.026	0.0466 – 0.306*
**Death**	0.0000	0.0000	0.0000	1.0000
**Decellularised allograft**				
	**To**			
**From**	**1st revision**	**Post-revision**	**Re-revision**	**Death**
**1st revision**	0.0000	0.688 – 0.947*	0.0064	0.0466 – 0.306*
**Post-revision**	0.0000	0.629 – 0.974*	0.0064	0.0195 – 0.364*
**Re-revision**	0.0000	0.688 – 0.947*	0.0064	0.0466 – 0.306*
**Death**	0.0000	0.0000	0.0000	1.0000

Supplementary Table 4: Fresh-frozen allograft RHA studies: the revision rate and subsequent transition probability for acetabular impaction bone grafting during a RHA, Average: (mean ± SD)

Source	Graft used	Revision rate	Follow-up time (years)	Transition rate	Transition Probability
[6]	Fresh-frozen morselised femoral head allograft	11.90%	10	0.013	0.013
[7]	Morselised allograft	20.00%	12	0.019	0.018
[8]	Morselised allograft	48.00%	25	0.026	0.026
[9]	Morselised femoral head allograft	25.00%	20	0.014	0.014
[10]	Bulky femoral head allograft	26.00%	10	0.03	0.03
[11]	Bulky femoral head allograft	12.50%	11.7	0.011	0.011
[12]	Bulky femoral head allograft	55.00%	20	0.04	0.039
[13]	Irradiated femoral head allograft	16.70%	10	0.018	0.018
[14]	Standard structural frozen-irradiated allograft	30.00%	12	0.03	0.029
				**Average:**	0.026 ± 0.017

Supplementary Table 5: Variable parameters used in stochastic model: The mean, standard deviation and distribution type used for each uncertain parameter.

Description	Graft	Distribution	Mean	SD	Sources

Probability of re-revision from revision	Fresh-frozen	Beta	0.026	0.017	Supplementary Table 3
	Decellularised	Fixed	0.0064	N/A	Supplementary Table 3
Probability of re-revision from success	Fresh-frozen	Beta	0.026	0.017	Supplementary Table 3
	Decellularised	Fixed	0.0064	N/A	Supplementary Table 3
Revision Utility	Both	Beta	0.397	0.354	[15]
Re-revision Utility	Both	Beta	0.397	0.354	[15]
Success Utility	Both	Beta	0.685	0.289	[15]
Mean age of cohort	Common	Log Normal	72.70	11.16	[1]
Inpatient stay cost (£)	Both	Gamma	£5230	£12038	[2]
Investigation costs	Both	Gamma	£523	£312	[3]
Drug costs	Both	Gamma	£398	£197	[3]
Implant costs	Both	Gamma	£2915	£3388	[3]
Theatre costs	Both	Gamma	£1389	£1132	[3]
Inpatient costs	Both	Gamma	£191	£558	[4]
Out-patient costs	Both	Gamma	£276	£210	[4]
Aids and adaptations	Both	Gamma	£21	£40	[4]
Medication	Both	Gamma	£24	£41	[4]
Reagents	Decellularised	Gamma	£591	£300	Supplementary Table 2
Lab rent	Decellularised	Gamma	£220	£78	[16, 17]

Supplementary References

[1] ONS, "GDP deflators at market prices, and money GDP March 2022 (Quarterly National Accounts)," ed, 2022.

[2] A. Webb, "Efficiency. The long goodbye," *The Health service journal,* vol. 118, pp. 26-7, 2008.

[3] I. S. Vanhegan, A. K. Malik, P. Jayakumar, S. Ul Islam, and F. S. Haddad, "A financial analysis of revision hip arthroplasty: the economic burden in relation to the national tariff," *The Journal of bone and joint surgery. British volume,* vol. 94, no. 5, pp. 619–623-619–623, 2012.

[4] R. Edlin, S. Tubeuf, J. Achten, N. Parsons, and M. Costa, "Cost-effectiveness of total hip arthroplasty versus resurfacing arthroplasty: economic evaluation alongside a clinical trial," *BMJ open,* vol. 2, no. 5, 2012.

[5] V. J. J. F. Busch, J. Verschueren, E. M. Adang, S. A. Lie, L. I. Havelin, and B. W. Schreurs, "A cemented cup with acetabular impaction bone grafting is more cost-effective than an uncemented cup in patients under 50 years," *Hip International,* vol. 26, no. 1, pp. 43–49-43–49, 2016.

[6] J. M. Warnock, C. H. Rowan, H. Davidson, C. Millar, and M. G. McAlinden, "Improving efficiency of a regional stand alone bone bank," *Cell and tissue banking,* vol. 17, no. 1, pp. 85–90-85–90, 2016.

[7] B. W. Schreurs *et al.*, "Acetabular revision with impacted morselized cancellous bone graft and a cemented cup in patients with rheumatoid arthritis: a concise follow-up, at eight to nineteen years, of a previous report," *JBJS,* vol. 91, no. 3, pp. 646–651-646–651, 2009.

[8] V. J. J. F. Busch, J. W. M. Gardeniers, N. Verdonschot, T. J. J. H. Slooff, and B. W. Schreurs, "Acetabular reconstruction with impaction bone-grafting and a cemented cup in patients younger than fifty years old: a concise follow-up, at twenty to twenty-eight years, of a previous report," *JBJS,* vol. 93, no. 4, pp. 367–371-367–371, 2011.

[9] B. W. Schreurs, J. C. Keurentjes, J. W. M. Gardeniers, N. Verdonschot, T. J. J. H. Slooff, and R. P. H. Veth, "Acetabular revision with impacted morsellised cancellous bone grafting and a cemented acetabular component: a 20-to 25-year follow-up," *The Journal of Bone and Joint Surgery. British volume,* vol. 91, no. 9, pp. 1148–1153-1148–1153, 2009.

[10] S. M. Sporer, M. O'Rourke, P. Chong, and W. G. Paprosky, "The use of structural distal femoral allografts for acetabular reconstruction: average ten-year follow-up," *JBJS,* vol. 87, no. 4, pp. 760-765, 2005.

[11] D. Regis, B. Magnan, A. Sandri, and P. Bartolozzi, "Long-term results of anti-protrusio cage and massive allografts for the management of periprosthetic acetabular bone loss," *The Journal of arthroplasty,* vol. 23, no. 6, pp. 826–832-826–832, 2008.

[12] P. T. H. Lee, G. Raz, O. A. Safir, D. J. Backstein, and A. E. Gross, "Long-term results for minor column allografts in revision hip arthroplasty," *Clinical Orthopaedics and Related Research®,* vol. 468, no. 12, pp. 3295–3303-3295–3303, 2010.

[13] N. W. Emms, S. C. Buckley, I. Stockley, A. J. Hamer, and R. M. Kerry, "Mid-to long-term results of irradiated allograft in acetabular reconstruction: a follow-up report," *The Journal of bone and joint surgery. British volume,* vol. 91, no. 11, pp. 1419–1423-1419–1423, 2009.

[14] P. Hernigou *et al.*, "Supercharging irradiated allografts with mesenchymal stem cells improves acetabular bone grafting in revision arthroplasty," *International orthopaedics,* vol. 38, no. 9, pp. 1913–1921-1913–1921, 2014.

[15] NHS Digital, "Provisional Monthly HES for Admitted Patient Care, Outpatient and Accident and Emergency Data - April 2014 - August 2014:Topic of Interest - Linked HES-ONS mortality data - 4 character procedure tables," ed, 2014.

[16] E. Slifer. "How to Determine If Your Lab is the Correct Size." https://www.linkedin.com/pulse/how-determine-your-lab-correct-size-eric-slifer/ (accessed 28/06/2022, 2022).

[17] Statista Research Department. "Average monthly rent of laboratory and life sciences space in the leading science cities worldwide in 2020, by city." https://www.statista.com/statistics/1192071/lab-space-rent-in-leading-science-cities-globally/#:~:text=These%20cities%20have%20the%20highest,U.S.%20dollars%20as%20of%202020. (accessed 28/06/2022, 2022).
