# Supplementary material for: Cost-effectiveness of decellularised bone allograft compared with fresh-frozen bone allograft for acetabular impaction bone grafting during a revision hip arthroplasty in the UK
Source: BMJ Open. 2023 Oct 6;13(10):e067876. doi: 10.1136/bmjopen-2022-067876 (PMC10565200; doi:10.1136/bmjopen-2022-067876)
Supplement: Author's manuscript [file bmjopen-2022-067876.draft_revisions.pdf]

BMJ Open is committed to open peer review. As part of this commitment we make the peer review history of every article we publish publicly available.

When an article is published we post the peer reviewers' comments and the authors' responses online. We also post the versions of the paper that were used during peer review. These are the versions that the peer review comments apply to.

The versions of the paper that follow are the versions that were submitted during the peer review process. They are not the versions of record or the final published versions. They should not be cited or distributed as the published version of this manuscript.

BMJ Open is an open access journal and the full, final, typeset and author-corrected version of record of the manuscript is available on our site with no access controls, subscription charges or pay-per-view fees (http://bmjopen.bmj.com).

If you have any questions on BMJ Open's open peer review process please email info.bmjopen@bmj.com

BMJ Open

Cost-effectiveness analysis of decellularised bone allograft compared with fresh-frozen bone allograft for acetabular impaction bone grafting during a revision hip arthroplasty

Journal:	*BMJ Open*
Manuscript ID	bmjopen-2022-067876
Article Type:	Original research
Date Submitted by the Author:	31-Aug-2022

Complete List of Authors:	Cowell, Kern; University of Leeds Faculty of Engineering<br>Sagoo, Gurdeep; University of Leeds, Academic Unit of Health Economics<br>Statham, Patrick; University of Leeds Faculty of Engineering<br>Chandler, James; University of Leeds Faculty of Engineering<br>Herbert, Anthony; University of Leeds Faculty of Engineering<br>Rooney, Paul; NHS Blood and Transplant Tissue and Eye Services<br>Wilcox, Ruth; University of Leeds Faculty of Engineering<br>Fermor, Hazel; University of Leeds Faculty of Biological Sciences

Keywords:	Hip < ORTHOPAEDIC & TRAUMA SURGERY, HEALTH ECONOMICS, Orthopaedic & trauma surgery < SURGERY, Bone diseases < ORTHOPAEDIC & TRAUMA SURGERY

SCHOLARONE™
Manuscripts

I, the Submitting Author has the right to grant and does grant on behalf of all authors of the Work (as defined in the below author licence), an exclusive licence and/or a non-exclusive licence for contributions from authors who are: i) UK Crown employees; ii) where BMJ has agreed a CC-BY licence shall apply, and/or iii) in accordance with the terms applicable for US Federal Government officers or employees acting as part of their official duties; on a worldwide, perpetual, irrevocable, royalty-free basis to BMJ Publishing Group Ltd ("BMJ") its licensees and where the relevant Journal is co-owned by BMJ to the co-owners of the Journal, to publish the Work in this journal and any other BMJ products and to exploit all rights, as set out in our licence.

The Submitting Author accepts and understands that any supply made under these terms is made by BMJ to the Submitting Author unless you are acting as an employee on behalf of your employer or a postgraduate student of an affiliated institution which is paying any applicable article publishing charge ("APC") for Open Access articles. Where the Submitting Author wishes to make the Work available on an Open Access basis (and intends to pay the relevant APC), the terms of reuse of such Open Access shall be governed by a Creative Commons licence – details of these licences and which Creative Commons licence will apply to this Work are set out in our licence referred to above.

Other than as permitted in any relevant BMJ Author's Self Archiving Policies, I confirm this Work has not been accepted for publication elsewhere, is not being considered for publication elsewhere and does not duplicate material already published. I confirm all authors consent to publication of this Work and authorise the granting of this licence.

For peer review only

Cost-effectiveness analysis of decellularised bone allograft compared with fresh-frozen bone allograft for acetabular impaction bone grafting during a revision hip arthroplasty

Kern Cowell, MEng [a], Gurdeep S Sagoo, PhD [b], Patrick Statham, BSc [a], James Chandler, PhD [c], Anthony Herbert, PhD [a], Paul Rooney PhD [e], Ruth Wilcox, PhD [a], Hazel L Fermor, PhD [d*]

[a] Institute of Medical and Biological Engineering, Faculty of Engineering and Physical Sciences, University of Leeds, Leeds, UK

[b] Academic Unit of Health Economics, Institute of Health Sciences, University of Leeds, Leeds, UK

[c] Institute of Design, Robotics and Optimisation, Faculty of Engineering and Physical Sciences, University of Leeds, Leeds, UK

[d] Institute of Medical and Biological Engineering, Faculty of Biological Sciences, University of Leeds, Leeds, UK

[e] NHS Blood and Transplant, Tissue and Eye Services, Speke, Liverpool

Corresponding author contact details: Dr Hazel Fermor, 6.57 Garstang Building, Faculty of Biological Sciences, University of Leeds, Leeds, LS2 9JT, UK; H.L.Fermor@leeds.ac.uk; +44 (0)113 3435651

Keywords: HEALTH ECONOMICS, Hip, Orthopaedic & trauma surgery, Bone diseases

Word count: 2479

For peer review only

Abstract

Aims: Fresh-frozen allograft is the gold standard bone graft material used during revision hip arthroplasty. However, new technology has been developed to manufacture decellularised bone with potentially better graft incorporation. As these grafts cost more to manufacture, this cost-effectiveness study was performed to estimate whether the potential health benefit of decellularised bone allograft outweighs their increased cost.

Methods: A Markov model was constructed to estimate the costs and the quality adjusted life years of impaction bone grafting during a revision hip arthroplasty, using either decellularised bone allograft or fresh-frozen allograft. Incremental cost-effectiveness ratios were calculated for both graft types using base case parameters and univariate sensitivity analysis.

Results: The incremental cost-effectiveness ratio for the base case model was found to be £212,181 per quality adjusted life years. Univariate sensitivity analysis found that changing the discount rate, the decellularised bone graft cost, age of the patient cohort, and the revision rate all had a significant effect on the incremental cost-effectiveness ratio.

Conclusions: As there are no clinical studies of impaction bone grafting using a decellularised bone allograft there is a high level of uncertainty around the costs of producing a decellularised bone allograft and the potential health benefits. However, if a decellularised bone graft was manufactured for £2847 and lowered the re-revision rate to less than 64 cases per year per 10,000 revision patients, then it would most likely be cost-effective compared to fresh-frozen allograft.

Strengths and Limitations

- The commercial cost of decellularising allograft bone has been calculated for the first time
- The cost-effectiveness of decellularised allograft bone versus fresh-frozen allograft bone has been evaluated for the first time
- This model and the costs determined for the decellularisation method could be replicated if future studies reveal a health benefit of using decellularised allograft bone over fresh-frozen allograft
- Due to a lack of clinical data on decellularised allograft bone, the revision rate of demineralised bone matrix in combination with allograft bone was assumed to be clinically equivalent which may not be accurate

Introduction

In the UK there are approximately 8000 revision hip arthroplasties (RHAs) each year [1], 28% of RHAs require an impaction bone graft (IBG) to replace removed bone before implantation of the joint components [2]. The current gold standard bone graft is an autograft [3]. However, as the average amount of bone tissue needed for an impaction bone graft is over two femoral heads per revision, allograft bone is most often used [2]. As allograft bone contains donor cellular material, there is a risk of a host immune reaction to the graft, which reduces regeneration, re-vascularisation and incorporation of the graft [4]. In the UK, 10% of revision hip arthroplasties require further revision [1], with the most

For peer review only

common reason being aseptic loosening (39% of cases) [5]. The use of an impaction bone graft during a RHA further increases the risk of aseptic loosening due to poor graft incorporation, infection and acetabular cup loosening [6]. If acetabular graft incorporation could be improved, then there is potential to increase the success of RHA and reduce the number of further revisions [7].

Decellularisation is a novel technology that removes cells and DNA from tissue, whilst preserving the underlying tissue matrix [8]. Depending on the manufacturing method used, decellularised bone allografts can retain the advantageous biological aspects of allografts such as osteoconductive growth factors and have similar mechanical properties to a fresh-frozen allograft, without evoking an adverse immune response [9]. These properties suggest that a decellularised impaction bone graft could have increased rates of regeneration and incorporation with the host environment, potentially decreasing the occurrence of aseptic loosening of hip replacement components [10].

However, with decellularisation of the allograft, there is an increase in the overall cost of the RHA (£22,674.36 per revision surgery). Therefore, any potential health benefit from decellularisation needs to be evaluated against the potential increase in cost. In this study a cost-effectiveness analysis was conducted to compare the novel decellularised allograft with the current most common bone graft option (fresh-frozen allograft) for acetabular impaction bone grafting during a RHA. The costs and health benefits of both bone graft choices were estimated and compared using a Markov model from the perspective of the UK National Health Service (NHS) [11].

Methods

Economic Modelling

A decision tree and Markov model [11] was constructed to estimate the costs and benefits of both graft choices (Figure 1). The model has yearly cycles that estimate the quality adjusted life years (QALYs) and costs over the lifetime of the patient from their first RHA shown by the decision tree. At year 0 of the model all patients start at the beginning of the decision tree at their first RHA, in the following years the patients transition between the 're-revision' and 'post-revision' health states or enter the 'death' health state. A discount rate of 3.5% per year for both cost and effectiveness was used in line with the recommendations from the National Institute for Health and Care Excellence (NICE) guide for technology appraisal [12]. The time spent in each health state was calculated and the weighted costs and QALYs of each health state were totalled to give the overall cost and QALYs for each bone graft choice.

To compare the two bone graft choices an incremental cost-effectiveness ratio (ICER) was used. The ICER is calculated by:

$$ICER = \frac{C_1 - C_0}{E_1 - E_0} \qquad (1)$$

Where C_1 is the cost and E_1 is the effectiveness of the new intervention with C_0 and E_0 being the cost and effectiveness of the original intervention [13]. In the UK, new treatment adoption decisions are guided by NICE through a technology appraisal which considers the ICER. If the ICER is below £20,000,

For peer review only

the new treatment is considered cost-effective, and its adoption is often recommended. An ICER of £20,000 - £30,000 indicates the need for more certainty around the estimated ICER or added benefits beyond the recorded QALYs e.g., life-extending treatment at the end of life. An ICER above £30,000 requires a substantial reason for the technology to be adopted e.g., it is an innovative technology that has benefits beyond the currently evaluated health benefits [12].

Costs

To find the cost of each bone graft choice, the cost of a RHA was combined with the cost of each bone graft (Tables 1 and 2). The individual costs of purchasing each graft were calculated for one femoral head, with the cost of 2.43 femoral head grafts used for each revision surgery (the average number used per procedure) [2].

Table 1: Cost of treatments: all costs are in £ (GBP) and converted into 2022 prices [14]

		Fresh-frozen allograft	Decellularised allograft	Source
Pre surgery	Inpatient stay cost (£)	6929.31		[15]
	Investigation costs	644.58		[16]
	Drug costs	490.84		[16]
Surgery	Implant costs	3593.18		[16]
	Theatre costs	1711.66		[16]
	Inpatient costs	235.44		[17]
	Impaction materials	617.50		[18]
	Total graft costs (2.43 femoral head grafts)	1724.59 [a]	24398.95 [b]	[a] [19]<br>[b] Table 2
Post-surgery costs	Out-patient costs	340.21		[17]
	Aids and adaptations	25.89		[17]
	Medication	29.58		[17]
**Total cost**		**16342.79**	**39017.15**	

Table 2: Price of decellularised femoral head allograft: all costs are in £ (GBP) and converted into 2022 prices [14], selling price based on average medical technology operating profit of 19.4% [20]

		Cost per graft	Sources
Product Cost	Tissue	709.71	[19]
	Reagents	557.56	Table 3
	Product labour	12.12	[21]
	Lab rent	219.75	[22, 23]

For peer review only

	Consumables and packaging	30.48	
	Amortised Investment	7.18	
Indirect Overheads		1536.80	
Sales and Distribution		5020.36	
**Selling Price**		**10040.72**	

The costs of the reagents used for the decellularisation process were obtained from a variety of suppliers (Table 3). The costs of the first revision surgery and the ‘re-revision’ health state were estimated under the assumption that the same bone impaction technique was used for each bone graft choice. The ‘post-revision’ health state has a cost of £54.19 per patient per year. This is representative of the average cost of rehabilitation, medication and other health service costs during the years after a RHA [24].

Table 3: Costs for each reagent used during decellularisation manufacturing: calculated from current suppliers. All costs are in £ (GBP) and converted into 2022 prices [14]

Reagent	Cost per graft
Aprotinin	85.34
Benzonase	238.69
Amphotericin B	6.57
Penicillin-Streptomycin	47.21
Phosphate-buffered saline	91.46
Nuclease Buffer	31.82
Hypertonic Buffer	31.82
Detergent Buffer	29.28
Hypotonic Buffer	29.28
**Total**	**591.47**

Utility

For this analysis, QALYs are used as the indicator of effectiveness and health benefit. QALYs incorporate the impact of a disease and treatment on both the quantity and quality of life and are the recommended outcome measure in the UK [12]. QALY values for the ‘re-revision’ and ‘post-revision’ health states were calculated from the pre-operative (‘re-revision’) and post-operative (‘post-revision’) patient reported outcome measures for revision total hip replacement [25]. Patients completed the EQ-5D-3L, a generic health-related quality of life measure, 6 months to a year after their RHA. As the patient reported outcome measures data only gives an overview of all revision hip replacement patients before and after their surgery, it was assumed in the base case analysis that the QALYs for the ‘re-revision’ (0.397) and ‘post-revision’ (0.685) health states for both bone graft choices were the same.

For peer review only

Transition probabilities

The transition probability is the probability that a patient will move from one health state to another during each period of the model (one year). Table 4 shows the transition probabilities used for both bone graft choices in the model.

Table 4: Transition Probabilities: Transition probabilities for all health states for both bone graft choices, *Dependent upon time period of model (year 1 to year 28 shown)

Fresh-frozen allograft				
	To			
**From**	**1st revision**	**Post-revision**	**Re-revision**	**Death**
**1st revision**	0.0000	0.668 – 0.927*	0.026	0.0466 – 0.306*
**Post-revision**	0.0000	0.061 – 0.954*	0.026	0.0195 – 0.364*
**Re-revision**	0.0000	0.6675 – 0.927*	0.026	0.0466 – 0.306*
**Death**	0.0000	0.0000	0.0000	1.0000
**Decellularised allograft**				
	To			
**From**	**1st revision**	**Post-revision**	**Re-revision**	**Death**
**1st revision**	0.0000	0.688 – 0.947*	0.0064	0.0466 – 0.306*
**Post-revision**	0.0000	0.629 – 0.974*	0.0064	0.0195 – 0.364*
**Re-revision**	0.0000	0.688 – 0.947*	0.0064	0.0466 – 0.306*
**Death**	0.0000	0.0000	0.0000	1.0000

It was assumed that the risk of needing a RHA is the same for all health states for each bone graft choice. For the fresh-frozen allograft the risk of re-revision was calculated from the studies summarised in Table 5 (clinical studies that include acetabular impaction bone grafting with a minimum follow-up period of 10 years). The transition rate (T_R) and transition probabilities (T_P) were calculated by:

$$T_R = \frac{-\ln(1 - Revision\ Rate\ (\%))}{Follow-up\ time\ (years)} \qquad T_P = 1 - e^{-T_R} \qquad (2)$$

Table 5: Fresh-frozen allograft RHA studies: the revision rate and subsequent transition probability for acetabular impaction bone grafting during a RHA, Average: (mean ± SD)

Source	Graft used	Revision rate	Follow-up time (years)	Transition rate	Transition Probability
[6]	Fresh-frozen morselised femoral head allograft	11.90%	10	0.013	0.013
[26]	Morselised allograft	20.00%	12	0.019	0.018
[27]	Morselised allograft	48.00%	25	0.026	0.026

For peer review only

[28]	Morselised femoral head allograft	25.00%	20	0.014	0.014
[29]	Bulky femoral head allograft	26.00%	10	0.03	0.03
[30]	Bulky femoral head allograft	12.50%	11.7	0.011	0.011
[31]	Bulky femoral head allograft	55.00%	20	0.04	0.039
[32]	Irradiated femoral head allograft	16.70%	10	0.018	0.018
[33]	Standard structural frozen-irradiated allograft	30.00%	12	0.03	0.029
				**Average:**	0.026 ± 0.017

To date, there have not yet been any clinical studies using decellularised bone as an impaction bone graft during RHA [10]. Therefore, to calculate the transition probabilities of re-revision, studies that tested demineralised bone in combination with allograft bone, that had a minimum follow-up period of 10 years were chosen. These criteria were chosen as adding demineralised bone matrix to allograft bone reduces the stiffness of the graft but increases the concentration of growth factors. For the purposes of this study, it was postulated that properties of demineralised bone matrix in combination with allograft bone would be similar to the properties of a decellularised bone graft. Only one study with this criterion was found in the literature, Hamadouche *et al.* [34] recorded an 8% revision rate for an impaction bone graft of demineralised bone matrix with allograft in 60 RHA patients after a 13-year follow-up. This gave a transition probability of 0.0064 for the 're-revision' health state. Another study with a follow-up of 2.25 years was also found [35], however the majority of re-revisions occurred in the first two years [5]. Therefore, this study was not included in this cost-effectiveness analysis as a short follow-up period can significantly over-estimate the calculated transition probability.

The transition from the health states to the 'death' state is based on the mean age of the patients in the model. The starting age used for the model was 71 years, this is the average age of RHA patients in the UK National Joint Registry [1]. A national life table was used to estimate the yearly transition probability for each year of the model [36]. As the base case model had an average starting age of 71.7 years, the transition probability from the 'post-revision' health state to the 'death' state started at 0.0195 for the first year and rose to 0.364 for year 29 of the model (subsequent years above patient age of 100 had a transition probability of 1). Due to the surgical risk and hospital care associated with a RHA, the transition probability for the first revision and' re-revision' health states to the death state were different for the first 90 days from the date of surgery, this changed the yearly transition probability to 0.047 for year 1 to 0.306 for year 29 of the model [37]. The combination of transition probabilities from each health state must equal 1. Therefore, the 'post-revision' transition probabilities for each year were calculated by subtracting the probability of entering the 're-revision' and 'death' health states from 1.

For peer review only

Univariate sensitivity analyses

To test which of the parameters affect the results of the model, a univariate sensitivity analysis was used. One variable in the model was changed to an extreme value and a new ICER was calculated. As suggested in the NICE guide for technology appraisal [12], the discount rate can significantly affect long term economic models and diminishes long term benefits. Therefore, a sensitivity analysis with a lower discount rate of 1.5% was completed. Other parameters of substantial uncertainty that were tested using sensitivity analysis were: the age of patients receiving a RHA; yearly post-surgery care costs; and the rate of re-revision of the decellularised graft.

Patient and public involvement Patients or the public were not involved in the design, or conduct, or reporting, or dissemination plans of our research

Patient consent for publication Not applicable.

Results

Cost-effectiveness analysis

For the base case parameters in this model, the overall revision surgery costs for the decellularized allograft were £39,017 per patient and the costs for the fresh-frozen allograft were £16,343 per patient. The QALYs calculated for the decellurised allograft choice were 6.93 per patient with the fresh-frozen allograft providing 6.86 QALYs per patient. To determine the cost-effectiveness of treatment options, the total costs and health benefits of each graft choice model are compared. For the base values in this model, neither bone graft option was found to be dominant, with the decellularised allograft costing more but providing an increase in QALYs (equivalent to 28 days of perfect health over their lifetime). For the base model the ICER of decellularised allograft was calculated as £270,059 per QALY, which is £240,059 higher than the NICE £30,000 per QALY guidance [12].

Univariate sensitivity analyses

As can be seen in Table 6, the primary parameters that affected the ICER during the sensitivity analysis were the discount rate, average age of the patient cohort, and the revision rate. Whereas the cost of care during the 'post-revision' health state had little effect on the ICER.

Table 6: Univariate sensitivity analysis: The incremental costs, QALYs and subsequent ICER from changing one parameter in the model to an extreme value

Scenario	Incremental costs	Incremental QALYs	ICER (Cost per QALY)	Favoured intervention
Base Case	£20,834	0.077	£270,059	Fresh-frozen
Discount rate changed from 3.5% to 1.5%	£20,511	0.092	£223,491	Fresh-frozen
Low patient cohort age (Mean - SD): 60.54 years	£20,110	0.133	£151,109	Fresh-frozen
High patient cohort age (Mean + SD): 82.86	£21,584	0.035	£623,920	Fresh-frozen

For peer review only

Low post-revision care costs: £0 per year	£20,821	0.077	£269,896	Fresh-frozen
High post-revision care costs: £301 per year	£20,895	0.077	£270,859	Fresh-frozen
Same re-revision rate	£28,783	0.000	-	Fresh-frozen

Discussion

The aim of this study was to evaluate the cost effectiveness of using a decellularised bone allograft for impaction bone grafting during a RHA from the perspective of the NHS. For the NICE £30,000 per QALY guidance, fresh-frozen allograft is more likely to be cost-effective than the decellularised graft. However, Although the favoured bone graft choice stayed the same throughout the univariate sensitivity analysis there were large changes to the ICER when the patient cohort age was changed, indicating how substantial any potential uncertainty around the input parameter values could be.

Logically, if the cost of producing a decellularised graft was reduced, this would increase the probability of the graft being cost effective. The current model assumes a large cost of retrieval of the femoral head bone graft (£709 per graft [19]). If an already functioning human tissue provider was to adopt decellularisation of their bone graft products, then this cost could be significantly reduced. The current cost estimate is based on a manual lab based decellularisation system, a commercial operation to manufacture decellularised bone grafts could benefit from cost savings using a large-scale automated manufacturing system. Producing decellularised grafts of tibial and femoral condyle bone for alternative applications alongside femoral head bone grafts would further reduce the costs of a decellularised femoral head graft, with costs shared across different grafts. Similarly, the cost per graft could be further reduced if other tissues were concurrently decellularised using the same manufacturing system. An overhead cost that was also not included in this study is the cost of storing the bone grafts. As decellularised bone grafts are sterilised during manufacturing, the grafts can be stored at room temperature, whereas fresh-frozen allografts require storage at -80 °C. Additionally, once decellularised, human tissue grafts are not considered to be relevant material under the Human Tissue Act [38] and therefore do not require the additional costs of licensing and tracking management that fresh-frozen allografts require.

Like the uncertainty around the costing of the decellularised graft, the effectiveness of the decellularised graft has also been estimated using many assumptions. As there are currently no clinical studies evaluating the outcome of a decellularised bone graft for impaction bone grafting during a RHA [10] the probability of requiring a further revision was estimated from a study of demineralised bone matrix with allograft bone [34]. However, decellularised bone grafts have better mechanical properties than demineralised bone matrix and allograft bone for high load applications and have reduced immunogenicity [39]. Therefore, the model used in this cost-effectiveness analysis could have underestimated the health benefit of the decellularised bone graft.

An alternative to decellularised allograft is washed bone. Studies have found that allograft bone that undergoes a washing process to remove bone marrow possesses similar mechanical properties to fresh-frozen allografts and the removal of immunogenic material could minimise immune reaction and increase bone regeneration [40]. Washed bone products have been provided by NHSBTTES and used

For peer review only

by orthopaedic surgeons since 2000 [41]. As there have been no reports of negative outcomes with these products it can be assumed that they perform well, and their continued use suggests that washed bone has clinical benefits over traditional fresh-frozen allograft bone. The cost-effectiveness of washed bone has yet to be systematically evaluated and compared with fresh-frozen or decellularised allograft bone.

Conclusion

The results of this cost-effectiveness analysis suggest that using the current manual manufacturing method, decellularised bone grafts are unlikely to be cost-effective compared to the current fresh-frozen allograft choice. However, if the purchase price of a decellularised bone allograft was below £2887 (71.2% reduction of the current price) and the graft adequately lowered the re-revision rate of hip arthroplasty to 64 re-revisions per year per 10000 patients as estimated, the ICER would be below the lower NICE guideline of £30,000 per QALY and the graft likely to be cost-effective. In conclusion, the model-based economic evaluation undertaken in this study suggests that if the cost of manufacturing decellularised bone allograft could be reduced, this would warrant exploration into the effectiveness of these grafts through a randomised clinical trial of impaction bone grafting during RHA.

Contributors KC, JC, AH, RKW, PR, and HLF conceived the study and participated in its design and coordination. KC and PS performed the data collection. KC, PS and GSS performed the statistical analyses and interpretation and drafted the manuscript. All authors read, revised, and approved the final manuscript. KC and HLF accept full responsibility for the work and/or the conduct of the study, had access to the data, and controlled the decision to publish.

Competing interests None declared.

Funding This study is funded by the Engineering and Physical Sciences Research Council (No. EP/L014823/1).

Disclaimer The funder had no role in the design and conduct of the study; collection, management, analysis, and interpretation of the data; preparation, review, or approval of the manuscript; and decision to submit the manuscript for publication

Data sharing statement The data associated with this paper (Costs of decellularisation and Markov model) are openly available from the University of Leeds Data Repository (DOI:to be requested on acceptance of paper publishing).

Ethics approval This was a modelling study and ethical approval from the institutional review board was not obtained because no real human participants or animal subjects were involved.

References

For peer review only

[1] NJR, "16th Annual Report," *National Joint Registry,* 2019.

[2] R. Lomas, A. Chandrasekar, and T. N. Board, "Bone allograft in the U.K.: perceptions and realities," *Hip Int,* https://doi.org/10.5301/hipint.5000018 vol. 23, no. 5, pp. 427-33, 2013.

[3] E. Benninger, P. O. Zingg, A. F. Kamath, and C. Dora, "Cost analysis of fresh-frozen femoral head allografts," *Bone Joint J,* vol. 96-B, pp. 1307–11, 2014, doi: https://doi.org/10.1302/0301-620X.96B10.33486.

[4] C. W. Cheng, L. D. Solorio, and E. Alsberg, "Decellularized tissue and cell-derived extracellular matrices as scaffolds for orthopaedic tissue engineering," *Biotechnol Adv,* https://doi.org/10.1016/j.biotechadv.2013.12.012 vol. 32, no. 2, pp. 462-84, 2014.

[5] A. H. Goldman, R. J. Sierra, R. T. Trousdale, D. G. Lewallen, D. J. Berry, and M. P. Abdel, "The Lawrence D. Dorr surgical techniques & technologies award: why are contemporary revision total hip arthroplasties failing? An Analysis of 2500 Cases," *The Journal of arthroplasty,* vol. 34, no. 7, pp. S11–S16-S11–S16, 2019.

[6] L. Busanelli, F. Castagnini, B. Bordini, S. Stea, P. P. Calderoni, and A. Toni, "The biological acetabular reconstruction with bone allografts in hip revision arthroplasty," *Musculoskeletal surgery,* vol. 103, no. 2, pp. 173–179-173–179, 2019.

[7] E. H. Van Haaren, I. C. Heyligers, F. G. M. Alexander, and P. I. J. M. Wuisman, "High rate of failure of impaction grafting in large acetabular defects," *The Journal of bone and joint surgery. British volume,* vol. 89, no. 3, pp. 296–300-296–300, 2007.

[8] H. L. Fermor, S. L. Russell, S. Williams, J. Fisher, and E. Ingham, "Development and characterisation of a decellularised bovine osteochondral biomaterial for cartilage repair," *J Mater Sci Mater Med,* https://doi.org/10.1007/s10856-015-5517-0 vol. 26, no. 5, pp. 186-186, 2015.

[9] C. Gardin *et al.*, "Decellularization and Delipidation Protocols of Bovine Bone and Pericardium for Bone Grafting and Guided Bone Regeneration Procedures," *PLoS One,* https://doi.org/10.1371/journal.pone.0132344 vol. 10, no. 7, pp. e0132344-e0132344, 2015.

[10] B. B. Rothrauff and R. S. Tuan, "Decellularized bone extracellular matrix in skeletal tissue engineering," *Biochemical Society Transactions,* pp. BST20190079-BST20190079, 2020.

[11] M. Komorowski and J. Raffa, "Markov models and cost effectiveness analysis: applications in medical research," in *Secondary analysis of electronic health records*: Springer, 2016, pp. 351–367-351–367.

[12] NICE, "Guide to the methods of technology appraisal 2013: 6 The appraisal of the evidence and structured decision-making," ed, 2013.

[13] J. Bhattacharya, T. Hyde, and P. Tu, *Health Economics*. Palgrave Macmillan, 2013.

[14] ONS, "GDP deflators at market prices, and money GDP March 2022 (Quarterly National Accounts)," ed, 2022.

[15] A. Webb, "Efficiency. The long goodbye," *The Health service journal,* vol. 118, pp. 26-7, 2008.

[16] I. S. Vanhegan, A. K. Malik, P. Jayakumar, S. Ul Islam, and F. S. Haddad, "A financial analysis of revision hip arthroplasty: the economic burden in relation to the national tariff," *The Journal of bone and joint surgery. British volume,* vol. 94, no. 5, pp. 619–623-619–623, 2012.

[17] R. Edlin, S. Tubeuf, J. Achten, N. Parsons, and M. Costa, "Cost-effectiveness of total hip arthroplasty versus resurfacing arthroplasty: economic evaluation alongside a clinical trial," *BMJ open,* vol. 2, no. 5, 2012.

[18] V. J. J. F. Busch, J. Verschueren, E. M. Adang, S. A. Lie, L. I. Havelin, and B. W. Schreurs, "A cemented cup with acetabular impaction bone grafting is more cost-effective than an uncemented cup in patients under 50 years," *Hip International,* vol. 26, no. 1, pp. 43–49-43–49, 2016.

For peer review only

[19] J. M. Warnock, C. H. Rowan, H. Davidson, C. Millar, and M. G. McAlinden, "Improving efficiency of a regional stand alone bone bank," *Cell and tissue banking,* vol. 17, no. 1, pp. 85–90-85–90, 2016.
[20] Mercer Capital, "Value Focus: Medtech and Device Industry," no. Fourth Quarter 2021, 2021.
[21] ONS, "Earnings and hours worked, occupation by two-digit SOC: ASHE Table 2; Provisional 2019," ed, 2019.
[22] E. Slifer. "How to Determine If Your Lab is the Correct Size." https://www.linkedin.com/pulse/how-determine-your-lab-correct-size-eric-slifer/ (accessed 28/06/2022, 2022).
[23] Statista Research Department. "Average monthly rent of laboratory and life sciences space in the leading science cities worldwide in 2020, by city." https://www.statista.com/statistics/1192071/lab-space-rent-in-leading-science-cities-globally/#:~:text=These%20cities%20have%20the%20highest,U.S.%20dollars%20as%20of%202020. (accessed 28/06/2022, 2022).
[24] N. Arden *et al.*, "Lower limb arthroplasty: can we produce a tool to predict outcome and failure, and is it cost-effective? An epidemiological study," *Programme Grants for Applied Research,* vol. 5, no. 12, pp. 1–246-1–246, 2017.
[25] NHS Digital, "Finalised Patient Reported Outcome Measures (PROMs) in England for Hip & Knee Replacements, April 2018 – March 2019," ed, 2019.
[26] B. W. Schreurs *et al.*, "Acetabular revision with impacted morselized cancellous bone graft and a cemented cup in patients with rheumatoid arthritis: a concise follow-up, at eight to nineteen years, of a previous report," *JBJS,* vol. 91, no. 3, pp. 646–651-646–651, 2009.
[27] V. J. J. F. Busch, J. W. M. Gardeniers, N. Verdonschot, T. J. J. H. Slooff, and B. W. Schreurs, "Acetabular reconstruction with impaction bone-grafting and a cemented cup in patients younger than fifty years old: a concise follow-up, at twenty to twenty-eight years, of a previous report," *JBJS,* vol. 93, no. 4, pp. 367–371-367–371, 2011.
[28] B. W. Schreurs, J. C. Keurentjes, J. W. M. Gardeniers, N. Verdonschot, T. J. J. H. Slooff, and R. P. H. Veth, "Acetabular revision with impacted morsellised cancellous bone grafting and a cemented acetabular component: a 20-to 25-year follow-up," *The Journal of Bone and Joint Surgery. British volume,* vol. 91, no. 9, pp. 1148–1153-1148–1153, 2009.
[29] S. M. Sporer, M. O'Rourke, P. Chong, and W. G. Paprosky, "The use of structural distal femoral allografts for acetabular reconstruction: average ten-year follow-up," *JBJS,* vol. 87, no. 4, pp. 760-765, 2005.
[30] D. Regis, B. Magnan, A. Sandri, and P. Bartolozzi, "Long-term results of anti-protrusio cage and massive allografts for the management of periprosthetic acetabular bone loss," *The Journal of arthroplasty,* vol. 23, no. 6, pp. 826–832-826–832, 2008.
[31] P. T. H. Lee, G. Raz, O. A. Safir, D. J. Backstein, and A. E. Gross, "Long-term results for minor column allografts in revision hip arthroplasty," *Clinical Orthopaedics and Related Research®,* vol. 468, no. 12, pp. 3295–3303-3295–3303, 2010.
[32] N. W. Emms, S. C. Buckley, I. Stockley, A. J. Hamer, and R. M. Kerry, "Mid-to long-term results of irradiated allograft in acetabular reconstruction: a follow-up report," *The Journal of bone and joint surgery. British volume,* vol. 91, no. 11, pp. 1419–1423-1419–1423, 2009.
[33] P. Hernigou *et al.*, "Supercharging irradiated allografts with mesenchymal stem cells improves acetabular bone grafting in revision arthroplasty," *International orthopaedics,* vol. 38, no. 9, pp. 1913–1921-1913–1921, 2014.
[34] M. Hamadouche, M. Karoubi, V. Dumaine, and J. P. Courpied, "The use of fibre-based demineralised bone matrix in major acetabular reconstruction: surgical technique and preliminary results," *International orthopaedics,* vol. 35, no. 2, pp. 283–288-283–288, 2011.

For peer review only

[35] G. Etienne, P. S. Ragland, and M. A. Mont, "Use of cancellous bone chips and demineralized bone matrix in the treatment of acetabular osteolysis: preliminary 2-year follow-up," *Orthopedics,* vol. 27, no. 1, pp. S123–S126-S123–S126, 2004.
[36] ONS, "National life tables, 2016-2018," ed, 2018.
[37] NHS Digital, "Provisional Monthly HES for Admitted Patient Care, Outpatient and Accident and Emergency Data - April 2014 - August 2014:Topic of Interest - Linked HES-ONS mortality data - 4 character procedure tables," ed, 2014.
[38] Human Tissue Act. "Human Tissue Act 2004." http://www.legislation.gov.uk/ukpga/2004/30/contents (accessed 22nd March 2021, 2021).
[39] M. Liu and Y. Lv, "Reconstructing bone with natural bone graft: a review of in vivo studies in bone defect animal model," *Nanomaterials,* vol. 8, no. 12, pp. 999-999, 2018.
[40] C. A. Smith, S. M. Richardson, M. J. Eagle, P. Rooney, T. Board, and J. A. Hoyland, "The use of a novel bone allograft wash process to generate a biocompatible, mechanically stable and osteoinductive biological scaffold for use in bone tissue engineering," *J Tissue Eng Regen Med,* https://doi.org/10.1002/term.1934 vol. 9, no. 5, pp. 595-604, 2015.
[41] M. J. Eagle, J. Man, P. Rooney, P. Hogg, and J. N. Kearney, "Assessment of an improved bone washing protocol for deceased donor human bone," *Cell Tissue Bank,* https://doi.org/10.1007/s10561-014-9443-z vol. 16, no. 1, pp. 83-90, 2015.

Figure 1

Decision tree and Markov model of post revision hip arthroplasty health states: Triangle end states show where decision tree enters Markov model; transition probabilities labelled as p_[to health state]_[from health state]

For peer review only

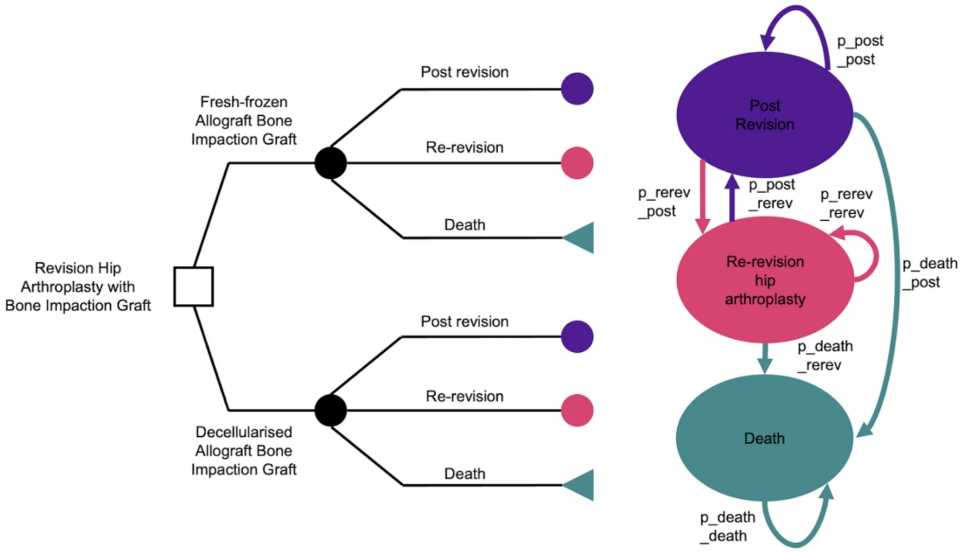

Decision tree and Markov model of post revision hip arthroplasty health states: Triangle end states show where decision tree enters Markov model; transition probabilities labelled as p_[to health state]_[from health state]

180x99mm (300 x 300 DPI)

CHEERS 2022 Checklist

Topic	No.	Item	Location where item is reported
**Title**			
	1	Identify the study as an economic evaluation and specify the interventions being compared.	Title, Page 2
**Abstract**			
	2	Provide a structured summary that highlights context, key methods, results, and alternative analyses.	Abstract, Page 3
**Introduction**			
**Background and objectives**	3	Give the context for the study, the study question, and its practical relevance for decision making in policy or practice.	Introduction, Paragraph 3, Page 4
**Methods**			
**Health economic analysis plan**	4	Indicate whether a health economic analysis plan was developed and where available.	Not Applicable
**Study population**	5	Describe characteristics of the study population (such as age range, demographics, socioeconomic, or clinical characteristics).	Methods: Economic Modelling, Paragraph 1, Page 4; Utility, Paragraph 1, Page 6; and Transition Probabilities, Paragraph 4, Page 8
**Setting and location**	6	Provide relevant contextual information that may influence findings.	Introduction, Paragraph 2, Page 4; Methods, Economic Modelling, Paragraph 1, Page 4
**Comparators**	7	Describe the interventions or strategies being compared and why chosen.	Introduction, Paragraph 2, Page 4
**Perspective**	8	State the perspective(s) adopted by the study and why chosen.	Introduction, Paragraph 2, Page 4
**Time horizon**	9	State the time horizon for the study and why appropriate.	Methods, Transition Probabilities, Paragraph 4, Page 8
**Discount rate**	10	Report the discount rate(s) and reason chosen.	Methods, Economic Modelling, Paragraph 1, Page 4

For peer review only - http://bmjopen.bmj.com/site/about/guidelines.xhtml

Topic	No.	Item	Location where item is reported
**Selection of outcomes**	11	Describe what outcomes were used as the measure(s) of benefit(s) and harm(s).	Methods, Utility, Paragraph 1, Page 6
**Measurement of outcomes**	12	Describe how outcomes used to capture benefit(s) and harm(s) were measured.	Methods, Utility, Paragraph 1, Page 6
**Valuation of outcomes**	13	Describe the population and methods used to measure and value outcomes.	Methods, Utility, Paragraph 1, Page 6
**Measurement and valuation of resources and costs**	14	Describe how costs were valued.	Methods, Costs, Page 5
**Currency, price date, and conversion**	15	Report the dates of the estimated resource quantities and unit costs, plus the currency and year of conversion.	Methods, Costs, Page 5
**Rationale and description of model**	16	If modelling is used, describe in detail and why used. Report if the model is publicly available and where it can be accessed.	Methods: Economic Modelling, Paragraph 1, Page 4; Transition Probabilities, Paragraph 3, Page 8
**Analytics and assumptions**	17	Describe any methods for analysing or statistically transforming data, any extrapolation methods, and approaches for validating any model used.	Methods: Economic Modelling, Page 4; Transition Probabilities, Page 7; Univariate Sensitivity Analyses, Page 9; and Results, Page 9
**Characterising heterogeneity**	18	Describe any methods used for estimating how the results of the study vary for subgroups.	Not applicable
**Characterising distributional effects**	19	Describe how impacts are distributed across different individuals or adjustments made to reflect priority populations.	Methods: Transition Probabilities, Page 7; and Univariate Sensitivity Analyses, Page 9
**Characterising uncertainty**	20	Describe methods to characterise any sources of uncertainty in the analysis.	Methods, Economic Modelling, Page 4; Transition Probabilities, Page 7; and Univariate Sensitivity Analyses, Page 9
**Approach to engagement with patients and others affected by the study**	21	Describe any approaches to engage patients or service recipients, the general public, communities, or stakeholders (such as clinicians or payers) in the design of the study.	Not reported

Topic	No.	Item	Location where item is reported
**Results**			
**Study parameters**	22	Report all analytic inputs (such as values, ranges, references) including uncertainty or distributional assumptions.	Methods: Costs, Tables 1 – 3, Page 5; Transition Probabilities, Tables 4 and 5, Page 7
**Summary of main results**	23	Report the mean values for the main categories of costs and outcomes of interest and summarise them in the most appropriate overall measure.	Results, Paragraph 1, Page 9
**Effect of uncertainty**	24	Describe how uncertainty about analytic judgments, inputs, or projections affect findings. Report the effect of choice of discount rate and time horizon, if applicable.	Results , Univariate Sensitivity Analyses, Page 9 and Discussion, Paragraphs 1 and 3, Page 10
**Effect of engagement with patients and others affected by the study**	25	Report on any difference patient/service recipient, general public, community, or stakeholder involvement made to the approach or findings of the study	Not reported
**Discussion**			
**Study findings, limitations, generalisability, and current knowledge**	26	Report key findings, limitations, ethical or equity considerations not captured, and how these could affect patients, policy, or practice.	Discussion, Page 10
**Other relevant information**			
**Source of funding**	27	Describe how the study was funded and any role of the funder in the identification, design, conduct, and reporting of the analysis	End of manuscript, Page 11
**Conflicts of interest**	28	Report authors conflicts of interest according to journal or International Committee of Medical Journal Editors requirements.	End of manuscript, Page 11

From: Husereau D, Drummond M, Augustovski F, et al. Consolidated Health Economic Evaluation Reporting Standards 2022 (CHEERS 2022) Explanation and Elaboration: A Report of the ISPOR CHEERS II Good Practices Task Force. Value Health 2022;25. doi:10.1016/j.jval.2021.10.008

For peer review only - http://bmjopen.bmj.com/site/about/guidelines.xhtml

BMJ Open

Cost-effectiveness of decellularised bone allograft compared with fresh-frozen bone allograft for acetabular impaction bone grafting during a revision hip arthroplasty in the UK

Journal:	*BMJ Open*
Manuscript ID	bmjopen-2022-067876.R1
Article Type:	Original research
Date Submitted by the Author:	19-Jul-2023
Complete List of Authors:	Cowell, Kern; University of Leeds Faculty of Engineering<br>Statham, Patrick; University of Leeds Faculty of Engineering<br>Sagoo, Gurdeep; University of Leeds, Academic Unit of Health Economics<br>Chandler, James; University of Leeds Faculty of Engineering<br>Herbert, Anthony; University of Leeds Faculty of Engineering<br>Rooney, Paul; NHS Blood and Transplant Tissue and Eye Services<br>Wilcox, Ruth; University of Leeds Faculty of Engineering<br>Fermor, Hazel; University of Leeds Faculty of Biological Sciences
**Primary Subject Heading**:	Health economics
Secondary Subject Heading:	Surgery
Keywords:	Hip < ORTHOPAEDIC & TRAUMA SURGERY, HEALTH ECONOMICS, Orthopaedic & trauma surgery < SURGERY, Bone diseases < ORTHOPAEDIC & TRAUMA SURGERY

SCHOLARONE™
Manuscripts

I, the Submitting Author has the right to grant and does grant on behalf of all authors of the Work (as defined in the below author licence), an exclusive licence and/or a non-exclusive licence for contributions from authors who are: i) UK Crown employees; ii) where BMJ has agreed a CC-BY licence shall apply, and/or iii) in accordance with the terms applicable for US Federal Government officers or employees acting as part of their official duties; on a worldwide, perpetual, irrevocable, royalty-free basis to BMJ Publishing Group Ltd ("BMJ") its licensees and where the relevant Journal is co-owned by BMJ to the co-owners of the Journal, to publish the Work in this journal and any other BMJ products and to exploit all rights, as set out in our licence.

The Submitting Author accepts and understands that any supply made under these terms is made by BMJ to the Submitting Author unless you are acting as an employee on behalf of your employer or a postgraduate student of an affiliated institution which is paying any applicable article publishing charge ("APC") for Open Access articles. Where the Submitting Author wishes to make the Work available on an Open Access basis (and intends to pay the relevant APC), the terms of reuse of such Open Access shall be governed by a Creative Commons licence – details of these licences and which Creative Commons licence will apply to this Work are set out in our licence referred to above.

Other than as permitted in any relevant BMJ Author's Self Archiving Policies, I confirm this Work has not been accepted for publication elsewhere, is not being considered for publication elsewhere and does not duplicate material already published. I confirm all authors consent to publication of this Work and authorise the granting of this licence.

For peer review only

Cost-effectiveness of decellularised bone allograft compared with fresh-frozen bone allograft for acetabular impaction bone grafting during a revision hip arthroplasty in the UK

Kern Cowell, PhD [a], Gurdeep S Sagoo, PhD [b], Patrick Statham, PhD [a], James Chandler, PhD [c], Anthony Herbert, PhD [a], Paul Rooney PhD [e], Ruth Wilcox, PhD [a], Hazel L Fermor, PhD [d]*

[a] Institute of Medical and Biological Engineering, Faculty of Engineering and Physical Sciences, University of Leeds, Leeds, UK

[b] Academic Unit of Health Economics, Institute of Health Sciences, University of Leeds, Leeds, UK

[c] Institute of Design, Robotics and Optimisation, Faculty of Engineering and Physical Sciences, University of Leeds, Leeds, UK

[d] Institute of Medical and Biological Engineering, Faculty of Biological Sciences, University of Leeds, Leeds, UK

[e] NHS Blood and Transplant, Tissue and Eye Services, Speke, Liverpool

Corresponding author contact details: Dr Hazel Fermor, 6.57 Garstang Building, Faculty of Biological Sciences, University of Leeds, Leeds, LS2 9JT, UK; H.L.Fermor@leeds.ac.uk; +44 (0)113 3435651

Keywords: HEALTH ECONOMICS, Hip, Orthopaedic & trauma surgery, Bone diseases

Word count: 3147

For peer review only

Abstract

Objectives Fresh-frozen allograft is the gold standard bone graft material used during revision hip arthroplasty. However, new technology has been developed to manufacture decellularised bone with potentially better graft incorporation. As these grafts cost more to manufacture, the aim of this cost-effectiveness study was to estimate whether the potential health benefit of decellularised bone allograft outweighs their increased cost.

Study design A Markov model was constructed to estimate the costs and the quality adjusted life years of impaction bone grafting during a revision hip arthroplasty.

Setting This study took the perspective of the NHS in the UK

Participants The Markov model includes patients undergoing a revision hip arthroplasty in the UK.

Intervention Impaction bone grafting during a revision hip arthroplasty using either decellularised bone allograft or fresh-frozen allograft.

Measures Outcome measures included: total costs and quality adjusted life years of both interventions over the lifetime of the model; and incremental cost-effectiveness ratios for both graft types, using base case parameters, univariate sensitivity analysis and probabilistic analysis.

Results The incremental cost-effectiveness ratio for the base case model was found to be £212,181 per quality adjusted life years. Univariate sensitivity analysis found that changing the discount rate, the decellularised bone graft cost, age of the patient cohort, and the revision rate all had a significant effect on the incremental cost-effectiveness ratio.

Conclusions As there are no clinical studies of impaction bone grafting using a decellularised bone allograft there is a high level of uncertainty around the costs of producing a decellularised bone allograft and the potential health benefits. However, if a decellularised bone graft was manufactured for £2847 and lowered the re-revision rate to less than 64 cases per year per 10,000 revision patients, then it would most likely be cost-effective compared to fresh-frozen allograft.

Strengths and Limitations

- By using the widely used Markov model, this study captured the dynamic nature of revision hip arthroplasty outcomes and estimated the costs and QALYs over the lifetime of the patient.
- The study included sensitivity analyses that assessed the variability and uncertainty of the findings by exploring the impact of key variables on the cost-effectiveness of the grafts used.
- The use of the stochastic modelling with 10,000 simulations provides a strong evaluation of the estimates made by accounting for parameter variability.
- One potential limitation is the lack of clinical data meaning the study relies on assumptions and extrapolation from studies involving different graft types for the decellularised graft revision rates.
- Another potential limitation is the use of costs from a variety of sources which may not accurately reflect the costs paid by the NHS in 2022.

For peer review only

Introduction

In the UK there are approximately 8000 revision hip arthroplasties (RHAs) each year [1], 28% of RHAs require an impaction bone graft (IBG) to replace removed bone before implantation of the joint components [2]. The current gold standard bone graft is an autograft [3]. However, as the average amount of bone tissue needed for an impaction bone graft is over two femoral heads per revision, allograft bone is most often used [2]. As allograft bone contains donor cellular material, there is a risk of a host immune reaction to the graft, which reduces regeneration, re-vascularisation and incorporation of the graft [4]. In the UK, 10% of revision hip arthroplasties require further revision [1], with the most common reason being aseptic loosening (39% of cases) [5]. The use of an impaction bone graft during a RHA further increases the risk of aseptic loosening due to poor graft incorporation, infection and acetabular cup loosening [6]. If acetabular graft incorporation could be improved, then there is potential to increase the success of RHA and reduce the number of further revisions [7].

Decellularisation is a novel technology that removes cells and DNA from tissue, whilst preserving the underlying tissue matrix [8]. Depending on the manufacturing method used, decellularised bone allografts can retain the advantageous biological aspects of allografts such as osteoconductive growth factors and have similar mechanical properties to a fresh-frozen allograft, without evoking an adverse immune response [9]. These properties suggest that a decellularised impaction bone graft could have increased rates of regeneration and incorporation with the host environment, potentially decreasing the occurrence of aseptic loosening of hip replacement components [10].

However, the overall costs of a RHA with decellularised allograft are £39017, compared with the overall cost of fresh-frozen allograft IBG: £16343, there is an increase of £22,674 per surgery. Therefore, any potential health benefit from decellularisation needs to be evaluated against the potential increase in cost. In this study a cost-effectiveness analysis was conducted to compare the novel decellularised allograft with the current most common bone graft option (fresh-frozen allograft) for acetabular impaction bone grafting during a RHA. The costs and health benefits of both bone graft choices were estimated and compared using a Markov model from the perspective of the UK National Health Service (NHS) [11].

Methods

Economic Modelling

A decision tree and Markov model [11] was constructed to estimate the costs and benefits of both graft choices (Figure 1). The model has yearly cycles that estimate the quality adjusted life years (QALYs) and costs over the lifetime of the patient from their first RHA shown by the decision tree. At year 0 of the model all patients start at the beginning of the decision tree at their first RHA, in the following years the patients transition between the 're-revision' and 'post-revision' health states or enter the 'death' health state. A discount rate of 3.5% per year for both cost and effectiveness was used in line with the recommendations from the National Institute for Health and Care Excellence (NICE) guide for technology appraisal [12]. The time spent in each health state was calculated and the weighted costs and QALYs of each health state were totalled to give the overall cost and QALYs for each bone graft choice.

For peer review only

To compare the two bone graft choices an incremental cost-effectiveness ratio (ICER) was used. The ICER is calculated by:

$$ICER = \frac{C_1 - C_0}{E_1 - E_0} \quad (1)$$

Where C_1 is the cost and E_1 is the effectiveness of the new intervention with C_0 and E_0 being the cost and effectiveness of the original intervention [13]. In the UK, new treatment adoption decisions are guided by NICE through a technology appraisal which considers the ICER. If the ICER is below £20,000, the new treatment is considered cost-effective, and its adoption is often recommended. An ICER of £20,000 - £30,000 indicates the need for more certainty around the estimated ICER or added benefits beyond the recorded QALYs e.g., life-extending treatment at the end of life. An ICER above £30,000 requires a substantial reason for the technology to be adopted e.g., it is an innovative technology that has benefits beyond the currently evaluated health benefits [12].

Costs

To find the cost of each bone graft choice, the cost of a RHA was combined with the cost of each bone graft (Table 1 and Supplementary Table 1). The individual costs of purchasing each graft were calculated for one femoral head, with the cost of 2.43 femoral head grafts used for each revision surgery (the average number used per procedure) [2]. As the NHS provides free public healthcare to residents of the UK, all estimated costs used in this study are those commonly paid by the NHS, including all inpatient stay costs, aids and adaptations and medication. The source used for the surgical costs did not include surgeon fees and therefore they were not included in this study [16]. Due to the same surgical time being used for both grafts, including surgeon fees would not change the results of the model.

Table 1: Price of decellularised femoral head allograft: all costs are in £ (GBP) and converted into 2022 prices [14], selling price based on average medical technology operating profit of 19.4% [15]

		Cost per graft	Sources
Product Cost	Tissue	709.71	[16]
	Reagents	557.56	Supplementary Table 2
	Product labour	12.12	[17]
	Lab rent	219.75	[18, 19]
	Consumables and packaging	30.48	
	Amortised Investment	7.18	
Indirect Overheads		1536.80	
Sales and Distribution		5020.36	
**Selling Price**		**10040.72**	

The costs of the reagents used for the decellularisation process were obtained from a variety of suppliers (Supplementary Table 2). The costs of the first revision surgery and the 're-revision' health

For peer review only

state were estimated under the assumption that the same bone impaction technique was used for each bone graft choice. The 'post-revision' health state has a cost of £54.19 per patient per year. This is representative of the average cost of rehabilitation, medication and other health service costs during the years after a RHA [20].

Utility

For this analysis, QALYs are used as the indicator of effectiveness and health benefit. QALYs incorporate the impact of a disease and treatment on both the quantity and quality of life and are the recommended outcome measure in the UK [12]. QALY values for the 're-revision' and 'post-revision' health states were calculated from the pre-operative ('re-revision') and post-operative ('post-revision') patient reported outcome measures for revision total hip replacement [21]. Patients completed the EQ-5D-3L, a generic health-related quality of life measure, 6 months to a year after their RHA. As the patient reported outcome measures data only gives an overview of all revision hip replacement patients before and after their surgery, it was assumed in the base case analysis that the QALYs for the 're-revision' (0.397) and 'post-revision' (0.685) health states for both bone graft choices were the same.

Transition probabilities

The transition probability is the probability that a patient will move from one health state to another during each period of the model (one year). Supplementary Table 3 shows the transition probabilities used for both bone graft choices in the model.

It was assumed that the risk of needing a RHA is the same for all health states for each bone graft choice. For the fresh-frozen allograft the risk of re-revision was calculated from the studies summarised in Supplementary Table 4 (clinical studies that include acetabular impaction bone grafting with a minimum follow-up period of 10 years). The transition rate (T_R) and transition probabilities (T_P) were calculated by:

$$T_R = \frac{-\ln(1 - Revision\ Rate\ (\%))}{Follow-up\ time\ (years)} \qquad T_P = 1 - e^{-T_R} \qquad (2)$$

To date, there have not yet been any clinical studies using decellularised bone as an impaction bone graft during RHA [10]. Therefore, to calculate the transition probabilities of re-revision, studies that tested demineralised bone in combination with allograft bone, that had a minimum follow-up period of 10 years were chosen. These criteria were chosen as adding demineralised bone matrix to allograft bone reduces the stiffness of the graft but increases the concentration of growth factors. For the purposes of this study, it was postulated that properties of demineralised bone matrix in combination with allograft bone would be similar to the properties of a decellularised bone graft. Only one study with this criterion was found in the literature, Hamadouche *et al.* [22] recorded an 8% revision rate for an impaction bone graft of demineralised bone matrix with allograft in 60 RHA patients after a 13-year follow-up. This gave a transition probability of 0.0064 for the 're-revision' health state. Another study with a follow-up of 2.25 years was also found [23], however the majority of re-revisions occurred in the

For peer review only

first two years [5]. Therefore, this study was not included in this cost-effectiveness analysis as a short follow-up period can significantly over-estimate the calculated transition probability.

The transition from the health states to the 'death' state is based on the mean age of the patients in the model. The starting age used for the model was 71 years, this is the average age of RHA patients in the UK National Joint Registry [1]. A national life table was used to estimate the yearly transition probability for each year of the model [24]. As the base case model had an average starting age of 71.7 years, the transition probability from the 'post-revision' health state to the 'death' state started at 0.0195 for the first year and rose to 0.364 for year 29 of the model (subsequent years above patient age of 100 had a transition probability of 1). Due to the surgical risk and hospital care associated with a RHA, the transition probability for the first revision and' re-revision' health states to the death state were different for the first 90 days from the date of surgery, this changed the yearly transition probability to 0.047 for year 1 to 0.306 for year 29 of the model [25]. The combination of transition probabilities from each health state must equal 1. Therefore, the 'post-revision' transition probabilities for each year were calculated by subtracting the probability of entering the 're-revision' and 'death' health states from 1.

Univariate sensitivity analyses

To test which of the parameters affect the results of the model, a univariate sensitivity analysis was used. One variable in the model was changed to an extreme value and a new ICER was calculated. As suggested in the NICE guide for technology appraisal [12], the discount rate can significantly affect long term economic models and diminishes long term benefits. Therefore, a sensitivity analysis with a lower discount rate of 1.5% was completed. Other parameters of substantial uncertainty that were tested using sensitivity analysis were: the age of patients receiving a RHA; yearly post-surgery care costs; and the rate of re-revision of the decellularised graft.

Stochastic model

Many of the parameters used in the Markov model have varying levels of uncertainty, therefore distributions of potential values were found using the mean and standard deviation of each parameter (Supplementary Table 5). To test the effect of this uncertainty on the ICER, 10,000 models were run using randomly generated values within each parameter distribution.

Patient and public involvement Patients or the public were not involved in the design, or conduct, or reporting, or dissemination plans of our research

Patient consent for publication Not applicable.

Results

Cost-effectiveness analysis

For the base case parameters in this model, the overall revision surgery costs for the decellularized allograft were £39,017 per patient and the costs for the fresh-frozen allograft were £16,343 per patient. The QALYs calculated for the decellurised allograft choice were 6.93 per patient with the fresh-frozen allograft providing 6.86 QALYs per patient. To determine the cost-effectiveness of treatment options,

For peer review only

the total costs and health benefits of each graft choice model are compared. For the base values in this model, neither bone graft option was found to be dominant, with the decellularised allograft costing more but providing an increase in QALYs (equivalent to 28 days of perfect health over their lifetime). For the base model the ICER of decellularised allograft was calculated as £270,059 per QALY, which is £240,059 higher than the NICE £30,000 per QALY guidance [12].

Univariate sensitivity analyses

As can be seen in Table 2, the primary parameters that affected the ICER during the sensitivity analysis were the discount rate, average age of the patient cohort, and the revision rate. Whereas the cost of care during the 'post-revision' health state had little effect on the ICER.

For peer review only

Table 2: Univariate sensitivity analysis: The incremental costs, QALYs and subsequent ICER from changing one parameter in the model to an extreme value

Scenario	Incremental costs	Incremental QALYs	ICER (Cost per QALY)	Favoured intervention
Base Case	£20,834	0.077	£270,059	Fresh-frozen
Discount rate changed from 3.5% to 1.5%	£20,511	0.092	£223,491	Fresh-frozen
Low patient cohort age (Mean - SD): 60.54 years	£20,110	0.133	£151,109	Fresh-frozen
High patient cohort age (Mean + SD): 82.86	£21,584	0.035	£623,920	Fresh-frozen
Low post-revision care costs: £0 per year	£20,821	0.077	£269,896	Fresh-frozen
High post-revision care costs: £301 per year	£20,895	0.077	£270,859	Fresh-frozen
Same re-revision rate	£28,783	0.000	-	Fresh-frozen

Stochastic Model

Calculated from the results of the randomly generated models, the percentage of models in which the decellularised graft was cheaper than fresh-frozen was 11.1% and the percentage in which the decellularised graft was more effective was 51.2%, this is highlighted in Figure 2 by the number of models below the x-axis (cheaper) and to the right of the y-axis (more effective). The average incremental cost and effectiveness of the models was found to be £20,620 and 0.089 QALYs. This gave an ICER estimate of £230,790 per QALY, £39,269 less per QALY than the initial base case model. The cost effectiveness acceptability curves in Figure 3 estimate the probability that one treatment is more cost effective than the other for different ICER values. The probability that the decellularised graft was cost-effective at the £30,000 per QALY threshold was 41.5% compared to 58.5% for fresh-frozen being cost-effective.

Discussion

The aim of this study was to evaluate the cost effectiveness of using a decellularised bone allograft for impaction bone grafting during a RHA from the perspective of the NHS. For the NICE £30,000 per QALY guidance, both base case and stochastic modelling indicated that fresh-frozen allograft is more likely to be cost-effective than the decellularised graft. However, although the favoured bone graft choice stayed the same throughout the univariate sensitivity analysis there were large changes to the ICER when the patient cohort age was changed, indicating how substantial any potential uncertainty around the input parameter values could be. Although, the stochastic model should effectively capture parameter uncertainty by collating the results of numerous simulations, enhancing the reliability of cost-effectiveness estimates.

Logically, if the cost of producing a decellularised graft was reduced, this would increase the probability of the graft being cost effective. The current model assumes a large cost of retrieval of the femoral head bone graft (£709 per graft [16]). If an already functioning human tissue provider was to adopt decellularisation of their bone graft products, then this cost could be significantly reduced. The current cost estimate is based on a manual lab based decellularisation system, a commercial operation to manufacture decellularised bone grafts could benefit from cost savings using a large-scale automated manufacturing system. Producing decellularised grafts of tibial and femoral condyle bone for alternative applications alongside femoral head bone grafts would further reduce the costs of a decellularised femoral head graft, with costs shared across different grafts. Similarly, the cost per graft could be further reduced if other tissues were concurrently decellularised using the same manufacturing system. An overhead cost that was also not included in this study is the cost of storing the bone grafts. As decellularised bone grafts are sterilised during manufacturing, the grafts can be stored at room temperature, whereas fresh-frozen allografts require storage at -80 °C. Additionally, once decellularised, human tissue grafts are not considered to be relevant material under the Human Tissue Act [26] and therefore do not require the additional costs of licensing and tracking management that fresh-frozen allografts require.

Like the uncertainty around the costing of the decellularised graft, the effectiveness of the decellularised graft has also been estimated using many assumptions. As there are currently no clinical studies evaluating the outcome of a decellularised bone graft for impaction bone grafting during a RHA [10] the probability of requiring a further revision was estimated from a study of demineralised bone matrix with allograft bone [22]. However, decellularised bone grafts have better mechanical properties than demineralised bone matrix and allograft bone for high load applications and have reduced immunogenicity [27]. Therefore, the model used in this cost-effectiveness analysis could have underestimated the health benefit of the decellularised bone graft.

Additionally, this study's utility measurement relies on QALYs estimated from generic revision hip arthroplasty patient data and does not indicate the specific health utility of impaction bone grafting patients for either graft type. Whilst this simplification was necessary due to a lack of direct clinical data, this could influence the results of the economic model. Furthermore, the study's cost assumptions are based on various sources from across different years, whilst the costs have been converted to 2022 GBP prices, these assumptions may not accurately reflect the 2022 costs spent by the NHS. Moreover,

For peer review only

the use of the good outcome care costs from Arden *et al.* [24] for the post-revision group in the model, assumes that any potential bad outcome of the RHA would require a re-revision, whereas a poor outcome could be possible that does not warrant revision but has more care costs. Although the sensitivity analysis (Table 2) shows any potential biases from this assumption have been shown to have a minor effect on cost-effectiveness.

An alternative to decellularised allograft is washed bone. Studies have found that allograft bone that undergoes a washing process to remove bone marrow possesses similar mechanical properties to fresh-frozen allografts and the removal of immunogenic material could minimise immune reaction and increase bone regeneration [28]. Washed bone products have been provided by NHSBTTES and used by orthopaedic surgeons since 2000 [29]. As there have been no reports of negative outcomes with these products it can be assumed that they perform well, and their continued use suggests that washed bone has clinical benefits over traditional fresh-frozen allograft bone. The cost-effectiveness of washed bone has yet to be systematically evaluated and compared with fresh-frozen or decellularised allograft bone.

Conclusion

The results of this cost-effectiveness analysis suggest that using the current manual manufacturing method, decellularised bone grafts are unlikely to be cost-effective compared to the current fresh-frozen allograft choice. However, if the purchase price of a decellularised bone allograft was below £2887 (71.2% reduction of the current price) and the graft adequately lowered the re-revision rate of hip arthroplasty to 64 re-revisions per year per 10000 patients as estimated, the ICER would be below the lower NICE guideline of £30,000 per QALY and the graft likely to be cost-effective. In conclusion, the model-based economic evaluation undertaken in this study suggests that if the cost of manufacturing decellularised bone allograft could be reduced, this would warrant exploration into the effectiveness of these grafts through a randomised clinical trial of impaction bone grafting during RHA.

Contributors KC, JC, AH, RKW, PR, and HLF conceived the study and participated in its design and coordination. KC and PS performed the data collection. KC, PS and GSS performed the statistical analyses and interpretation and drafted the manuscript. All authors read, revised, and approved the final manuscript. KC and HLF accept full responsibility for the work and/or the conduct of the study, had access to the data, and controlled the decision to publish.

Competing interests None declared.

Funding This study is funded by the Engineering and Physical Sciences Research Council (No. EP/L014823/1).

Disclaimer The funder had no role in the design and conduct of the study; collection, management, analysis, and interpretation of the data; preparation, review, or approval of the manuscript; and decision to submit the manuscript for publication

Data sharing statement Data available upon reasonable request

For peer review only

Ethics approval This was a modelling study and ethical approval from the institutional review board was not obtained because no real human participants or animal subjects were involved.

References

[1] NJR, "16th Annual Report," *National Joint Registry,* 2019.

[2] R. Lomas, A. Chandrasekar, and T. N. Board, "Bone allograft in the U.K.: perceptions and realities," *Hip Int,* https://doi.org/10.5301/hipint.5000018 vol. 23, no. 5, pp. 427-33, 2013.

[3] E. Benninger, P. O. Zingg, A. F. Kamath, and C. Dora, "Cost analysis of fresh-frozen femoral head allografts," *Bone Joint J,* vol. 96-B, pp. 1307–11, 2014, doi: https://doi.org/10.1302/0301-620X.96B10.33486.

[4] C. W. Cheng, L. D. Solorio, and E. Alsberg, "Decellularized tissue and cell-derived extracellular matrices as scaffolds for orthopaedic tissue engineering," *Biotechnol Adv,* https://doi.org/10.1016/j.biotechadv.2013.12.012 vol. 32, no. 2, pp. 462-84, 2014.

[5] A. H. Goldman, R. J. Sierra, R. T. Trousdale, D. G. Lewallen, D. J. Berry, and M. P. Abdel, "The Lawrence D. Dorr surgical techniques & technologies award: why are contemporary revision total hip arthroplasties failing? An Analysis of 2500 Cases," *The Journal of arthroplasty,* vol. 34, no. 7, pp. S11–S16-S11–S16, 2019.

[6] L. Busanelli, F. Castagnini, B. Bordini, S. Stea, P. P. Calderoni, and A. Toni, "The biological acetabular reconstruction with bone allografts in hip revision arthroplasty," *Musculoskeletal surgery,* vol. 103, no. 2, pp. 173–179-173–179, 2019.

[7] E. H. Van Haaren, I. C. Heyligers, F. G. M. Alexander, and P. I. J. M. Wuisman, "High rate of failure of impaction grafting in large acetabular defects," *The Journal of bone and joint surgery. British volume,* vol. 89, no. 3, pp. 296–300-296–300, 2007.

[8] H. L. Fermor, S. L. Russell, S. Williams, J. Fisher, and E. Ingham, "Development and characterisation of a decellularised bovine osteochondral biomaterial for cartilage repair," *J Mater Sci Mater Med,* https://doi.org/10.1007/s10856-015-5517-0 vol. 26, no. 5, pp. 186-186, 2015.

[9] C. Gardin *et al.*, "Decellularization and Delipidation Protocols of Bovine Bone and Pericardium for Bone Grafting and Guided Bone Regeneration Procedures," *PLoS One,* https://doi.org/10.1371/journal.pone.0132344 vol. 10, no. 7, pp. e0132344-e0132344, 2015.

[10] B. B. Rothrauff and R. S. Tuan, "Decellularized bone extracellular matrix in skeletal tissue engineering," *Biochemical Society Transactions,* pp. BST20190079-BST20190079, 2020.

[11] M. Komorowski and J. Raffa, "Markov models and cost effectiveness analysis: applications in medical research," in *Secondary analysis of electronic health records*: Springer, 2016, pp. 351–367-351–367.

[12] NICE, "Guide to the methods of technology appraisal 2013: 6 The appraisal of the evidence and structured decision-making," ed, 2013.

[13] J. Bhattacharya, T. Hyde, and P. Tu, *Health Economics*. Palgrave Macmillan, 2013.

[14] ONS, "GDP deflators at market prices, and money GDP March 2022 (Quarterly National Accounts)," ed, 2022.

[15] Mercer Capital, "Value Focus: Medtech and Device Industry," no. Fourth Quarter 2021, 2021.

[16] J. M. Warnock, C. H. Rowan, H. Davidson, C. Millar, and M. G. McAlinden, "Improving efficiency of a regional stand alone bone bank," *Cell and tissue banking,* vol. 17, no. 1, pp. 85–90-85–90, 2016.

[17] ONS, "Earnings and hours worked, occupation by two-digit SOC: ASHE Table 2; Provisional 2019," ed, 2019.

For peer review only

[18] E. Slifer. "How to Determine If Your Lab is the Correct Size." https://www.linkedin.com/pulse/how-determine-your-lab-correct-size-eric-slifer/ (accessed 28/06/2022, 2022).

[19] Statista Research Department. "Average monthly rent of laboratory and life sciences space in the leading science cities worldwide in 2020, by city." https://www.statista.com/statistics/1192071/lab-space-rent-in-leading-science-cities-globally/#:~:text=These%20cities%20have%20the%20highest,U.S.%20dollars%20as%20of%202020. (accessed 28/06/2022, 2022).

[20] N. Arden *et al.*, "Lower limb arthroplasty: can we produce a tool to predict outcome and failure, and is it cost-effective? An epidemiological study," *Programme Grants for Applied Research,* vol. 5, no. 12, pp. 1–246-1–246, 2017.

[21] NHS Digital, "Finalised Patient Reported Outcome Measures (PROMs) in England for Hip & Knee Replacements, April 2018 – March 2019," ed, 2019.

[22] M. Hamadouche, M. Karoubi, V. Dumaine, and J. P. Courpied, "The use of fibre-based demineralised bone matrix in major acetabular reconstruction: surgical technique and preliminary results," *International orthopaedics,* vol. 35, no. 2, pp. 283–288-283–288, 2011.

[23] G. Etienne, P. S. Ragland, and M. A. Mont, "Use of cancellous bone chips and demineralized bone matrix in the treatment of acetabular osteolysis: preliminary 2-year follow-up," *Orthopedics,* vol. 27, no. 1, pp. S123–S126-S123–S126, 2004.

[24] ONS, "National life tables, 2016-2018," ed, 2018.

[25] NHS Digital, "Provisional Monthly HES for Admitted Patient Care, Outpatient and Accident and Emergency Data - April 2014 - August 2014:Topic of Interest - Linked HES-ONS mortality data - 4 character procedure tables," ed, 2014.

[26] Human Tissue Act. "Human Tissue Act 2004." http://www.legislation.gov.uk/ukpga/2004/30/contents (accessed 22nd March 2021, 2021).

[27] M. Liu and Y. Lv, "Reconstructing bone with natural bone graft: a review of in vivo studies in bone defect animal model," *Nanomaterials,* vol. 8, no. 12, pp. 999-999, 2018.

[28] C. A. Smith, S. M. Richardson, M. J. Eagle, P. Rooney, T. Board, and J. A. Hoyland, "The use of a novel bone allograft wash process to generate a biocompatible, mechanically stable and osteoinductive biological scaffold for use in bone tissue engineering," *J Tissue Eng Regen Med,* https://doi.org/10.1002/term.1934 vol. 9, no. 5, pp. 595-604, 2015.

[29] M. J. Eagle, J. Man, P. Rooney, P. Hogg, and J. N. Kearney, "Assessment of an improved bone washing protocol for deceased donor human bone," *Cell Tissue Bank,* https://doi.org/10.1007/s10561-014-9443-z vol. 16, no. 1, pp. 83-90, 2015.

Figure 1

Decision tree and Markov model of post revision hip arthroplasty health states: Triangle end states show where decision tree enters Markov model; transition probabilities labelled as p_[to health state]_[from health state]

Figure 2

Stochastic model estimates: incremental costs and QALYs for each of the 10,000 randomly generated parameter models

Figure 3

For peer review only

Cost-effectiveness acceptability curves: the probability that each bone graft choice is cost-effective at varying willingness to pay thresholds

For peer review only

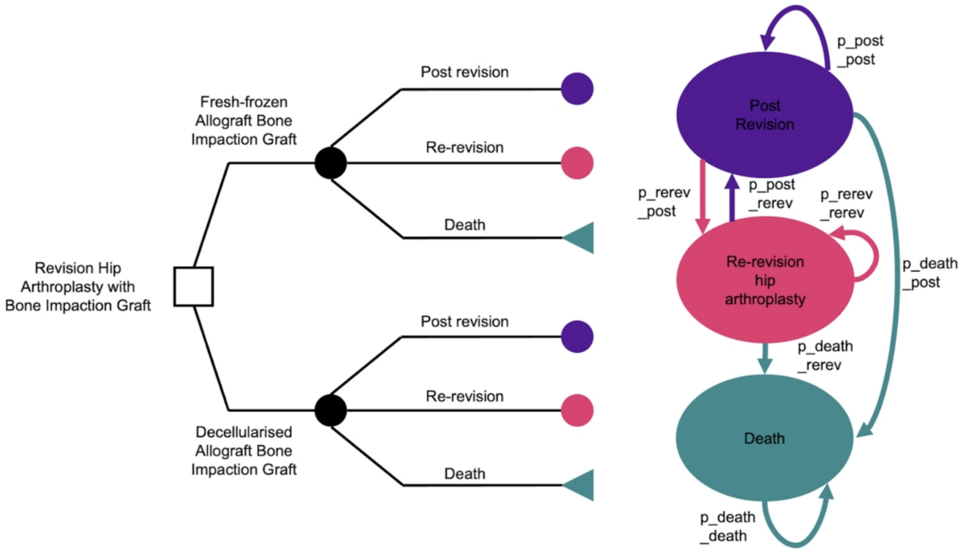

Decision tree and Markov model of post revision hip arthroplasty health states: Triangle end states show where decision tree enters Markov model; transition probabilities labelled as p_[to health state]_[from health state]

180x99mm (330 x 330 DPI)

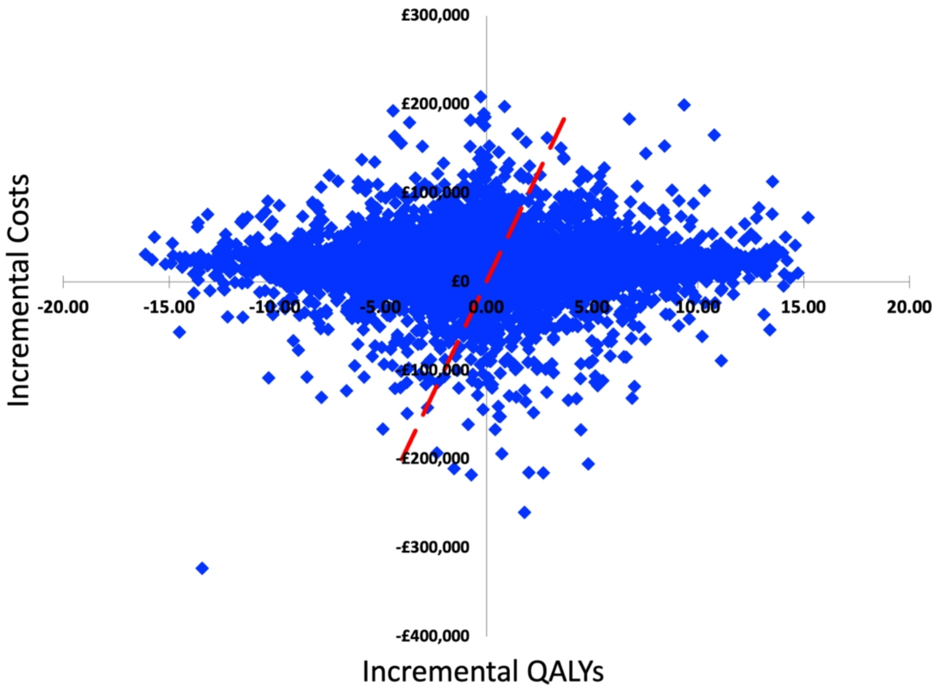

Stochastic model estimates: incremental costs and QALYs for each of the 10,000 randomly generated parameter models.

196x144mm (300 x 300 DPI)

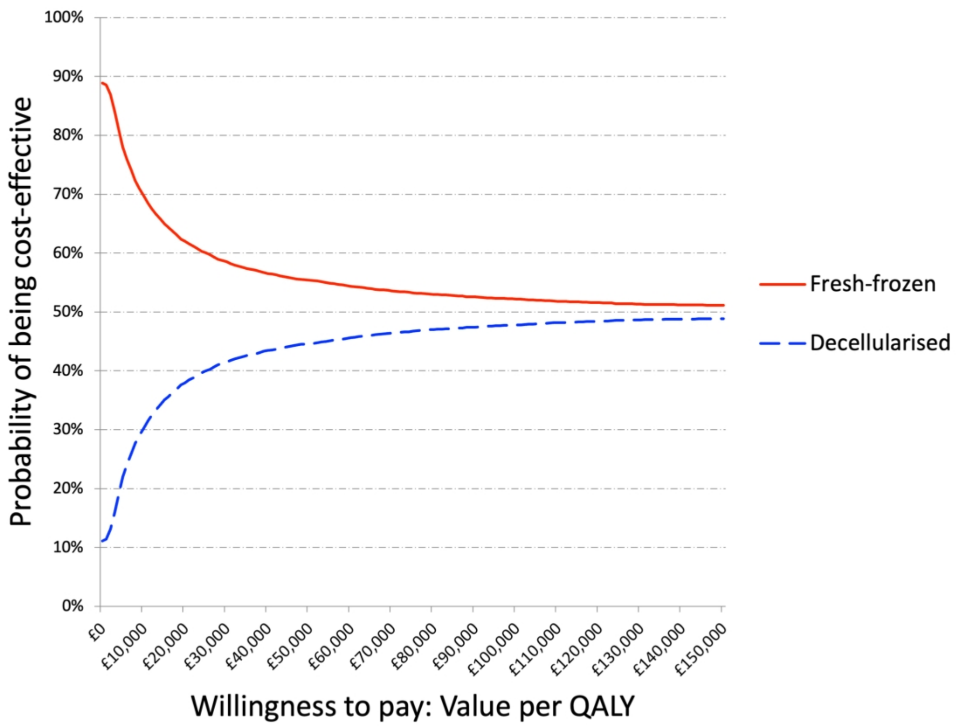

Cost-effectiveness acceptability curves: the probability that each bone graft choice is cost-effective at varying willingness to pay thresholds.

199x149mm (300 x 300 DPI)

Supplementary Table 1: Cost of treatments: all costs are in £ (GBP) and converted into 2022 prices [1]

		Fresh-frozen allograft	Decellularised allograft	Source
Pre surgery	Inpatient stay cost (£)	6929.31		[2]
	Investigation costs	644.58		[3]
	Drug costs	490.84		[3]
Surgery	Implant costs	3593.18		[3]
	Theatre costs	1711.66		[3]
	Inpatient costs	235.44		[4]
	Impaction materials	617.50		[5]
	Total graft costs (2.43 femoral head grafts)	1724.59 [a]	24398.95 [b]	[a] [6] [b] Table 1
Post-surgery costs	Out-patient costs	340.21		[4]
	Aids and adaptations	25.89		[4]
	Medication	29.58		[4]
**Total cost**		**16342.79**	**39017.15**	

Supplementary Table 2: Costs for each reagent used during decellularisation manufacturing: calculated from current suppliers. All costs are in £ (GBP) and converted into 2022 prices [1]

Reagent	Cost per graft
Aprotinin	85.34
Benzonase	238.69
Amphotericin B	6.57
Penicillin-Streptomycin	47.21
Phosphate-buffered saline	91.46
Nuclease Buffer	31.82
Hypertonic Buffer	31.82
Detergent Buffer	29.28
Hypotonic Buffer	29.28
**Total**	**591.47**

Supplementary Table 3: Transition Probabilities: Transition probabilities for all health states for both bone graft choices, *Dependent upon time period of model (year 1 to year 28 shown)

For peer review only

Fresh-frozen allograft				
	To			
From	1st revision	Post-revision	Re-revision	Death
1st revision	0.0000	0.668 – 0.927*	0.026	0.0466 – 0.306*
Post-revision	0.0000	0.061 – 0.954*	0.026	0.0195 – 0.364*
Re-revision	0.0000	0.6675 – 0.927*	0.026	0.0466 – 0.306*
Death	0.0000	0.0000	0.0000	1.0000
**Decellularised allograft**				
	To			
From	1st revision	Post-revision	Re-revision	Death
1st revision	0.0000	0.688 – 0.947*	0.0064	0.0466 – 0.306*
Post-revision	0.0000	0.629 – 0.974*	0.0064	0.0195 – 0.364*
Re-revision	0.0000	0.688 – 0.947*	0.0064	0.0466 – 0.306*
Death	0.0000	0.0000	0.0000	1.0000

Supplementary Table 4: Fresh-frozen allograft RHA studies: the revision rate and subsequent transition probability for acetabular impaction bone grafting during a RHA, Average: (mean ± SD)

Source	Graft used	Revision rate	Follow-up time (years)	Transition rate	Transition Probability
[6]	Fresh-frozen morselised femoral head allograft	11.90%	10	0.013	0.013
[7]	Morselised allograft	20.00%	12	0.019	0.018
[8]	Morselised allograft	48.00%	25	0.026	0.026
[9]	Morselised femoral head allograft	25.00%	20	0.014	0.014
[10]	Bulky femoral head allograft	26.00%	10	0.03	0.03
[11]	Bulky femoral head allograft	12.50%	11.7	0.011	0.011
[12]	Bulky femoral head allograft	55.00%	20	0.04	0.039
[13]	Irradiated femoral head allograft	16.70%	10	0.018	0.018
[14]	Standard structural frozen-irradiated allograft	30.00%	12	0.03	0.029
				**Average:**	0.026 ± 0.017

Supplementary Table 5: Variable parameters used in stochastic model: The mean, standard deviation and distribution type used for each uncertain parameter.

Description	Graft	Distribution	Mean	SD	Sources

Probability of re-revision from revision	Fresh-frozen	Beta	0.026	0.017	Supplementary Table 3
	Decellularised	Fixed	0.0064	N/A	Supplementary Table 3
Probability of re-revision from success	Fresh-frozen	Beta	0.026	0.017	Supplementary Table 3
	Decellularised	Fixed	0.0064	N/A	Supplementary Table 3
Revision Utility	Both	Beta	0.397	0.354	[15]
Re-revision Utility	Both	Beta	0.397	0.354	[15]
Success Utility	Both	Beta	0.685	0.289	[15]
Mean age of cohort	Common	Log Normal	72.70	11.16	[1]
Inpatient stay cost (£)	Both	Gamma	£5230	£12038	[2]
Investigation costs	Both	Gamma	£523	£312	[3]
Drug costs	Both	Gamma	£398	£197	[3]
Implant costs	Both	Gamma	£2915	£3388	[3]
Theatre costs	Both	Gamma	£1389	£1132	[3]
Inpatient costs	Both	Gamma	£191	£558	[4]
Out-patient costs	Both	Gamma	£276	£210	[4]
Aids and adaptations	Both	Gamma	£21	£40	[4]
Medication	Both	Gamma	£24	£41	[4]
Reagents	Decellularised	Gamma	£591	£300	Supplementary Table 2
Lab rent	Decellularised	Gamma	£220	£78	[16, 17]

Supplementary References

[1] ONS, "GDP deflators at market prices, and money GDP March 2022 (Quarterly National Accounts)," ed, 2022.

[2] A. Webb, "Efficiency. The long goodbye," *The Health service journal,* vol. 118, pp. 26-7, 2008.

[3] I. S. Vanhegan, A. K. Malik, P. Jayakumar, S. Ul Islam, and F. S. Haddad, "A financial analysis of revision hip arthroplasty: the economic burden in relation to the national tariff," *The Journal of bone and joint surgery. British volume,* vol. 94, no. 5, pp. 619–623-619–623, 2012.

[4] R. Edlin, S. Tubeuf, J. Achten, N. Parsons, and M. Costa, "Cost-effectiveness of total hip arthroplasty versus resurfacing arthroplasty: economic evaluation alongside a clinical trial," *BMJ open,* vol. 2, no. 5, 2012.

[5] V. J. J. F. Busch, J. Verschueren, E. M. Adang, S. A. Lie, L. I. Havelin, and B. W. Schreurs, "A cemented cup with acetabular impaction bone grafting is more cost-effective than an uncemented cup in patients under 50 years," *Hip International,* vol. 26, no. 1, pp. 43–49-43–49, 2016.

[6] J. M. Warnock, C. H. Rowan, H. Davidson, C. Millar, and M. G. McAlinden, "Improving efficiency of a regional stand alone bone bank," *Cell and tissue banking,* vol. 17, no. 1, pp. 85–90-85–90, 2016.

[7] B. W. Schreurs *et al.*, "Acetabular revision with impacted morselized cancellous bone graft and a cemented cup in patients with rheumatoid arthritis: a concise follow-up, at eight to nineteen years, of a previous report," *JBJS,* vol. 91, no. 3, pp. 646–651-646–651, 2009.

[8] V. J. J. F. Busch, J. W. M. Gardeniers, N. Verdonschot, T. J. J. H. Slooff, and B. W. Schreurs, "Acetabular reconstruction with impaction bone-grafting and a cemented cup in patients younger than fifty years old: a concise follow-up, at twenty to twenty-eight years, of a previous report," *JBJS,* vol. 93, no. 4, pp. 367–371-367–371, 2011.

For peer review only

[9] B. W. Schreurs, J. C. Keurentjes, J. W. M. Gardeniers, N. Verdonschot, T. J. J. H. Slooff, and R. P. H. Veth, "Acetabular revision with impacted morsellised cancellous bone grafting and a cemented acetabular component: a 20-to 25-year follow-up," *The Journal of Bone and Joint Surgery. British volume,* vol. 91, no. 9, pp. 1148–1153-1148–1153, 2009.

[10] S. M. Sporer, M. O'Rourke, P. Chong, and W. G. Paprosky, "The use of structural distal femoral allografts for acetabular reconstruction: average ten-year follow-up," *JBJS,* vol. 87, no. 4, pp. 760-765, 2005.

[11] D. Regis, B. Magnan, A. Sandri, and P. Bartolozzi, "Long-term results of anti-protrusio cage and massive allografts for the management of periprosthetic acetabular bone loss," *The Journal of arthroplasty,* vol. 23, no. 6, pp. 826–832-826–832, 2008.

[12] P. T. H. Lee, G. Raz, O. A. Safir, D. J. Backstein, and A. E. Gross, "Long-term results for minor column allografts in revision hip arthroplasty," *Clinical Orthopaedics and Related Research®,* vol. 468, no. 12, pp. 3295–3303-3295–3303, 2010.

[13] N. W. Emms, S. C. Buckley, I. Stockley, A. J. Hamer, and R. M. Kerry, "Mid-to long-term results of irradiated allograft in acetabular reconstruction: a follow-up report," *The Journal of bone and joint surgery. British volume,* vol. 91, no. 11, pp. 1419–1423-1419–1423, 2009.

[14] P. Hernigou *et al.*, "Supercharging irradiated allografts with mesenchymal stem cells improves acetabular bone grafting in revision arthroplasty," *International orthopaedics,* vol. 38, no. 9, pp. 1913–1921-1913–1921, 2014.

[15] NHS Digital, "Provisional Monthly HES for Admitted Patient Care, Outpatient and Accident and Emergency Data - April 2014 - August 2014:Topic of Interest - Linked HES-ONS mortality data - 4 character procedure tables," ed, 2014.

[16] E. Slifer. "How to Determine If Your Lab is the Correct Size." https://www.linkedin.com/pulse/how-determine-your-lab-correct-size-eric-slifer/ (accessed 28/06/2022, 2022).

[17] Statista Research Department. "Average monthly rent of laboratory and life sciences space in the leading science cities worldwide in 2020, by city." https://www.statista.com/statistics/1192071/lab-space-rent-in-leading-science-cities-globally/#:~:text=These%20cities%20have%20the%20highest,U.S.%20dollars%20as%20of%202020. (accessed 28/06/2022, 2022).

For peer review only

CHEERS 2022 Checklist

Topic	No.	Item	Location where item is reported
**Title**			
	1	Identify the study as an economic evaluation and specify the interventions being compared.	Title, Page 2
**Abstract**			
	2	Provide a structured summary that highlights context, key methods, results, and alternative analyses.	Abstract, Page 3
**Introduction**			
**Background and objectives**	3	Give the context for the study, the study question, and its practical relevance for decision making in policy or practice.	Introduction, Paragraph 3, Page 4
**Methods**			
**Health economic analysis plan**	4	Indicate whether a health economic analysis plan was developed and where available.	Not Applicable
**Study population**	5	Describe characteristics of the study population (such as age range, demographics, socioeconomic, or clinical characteristics).	Methods: Economic Modelling, Paragraph 1, Page 4; Utility, Paragraph 1, Page 6; and Transition Probabilities, Paragraph 4, Page 8
**Setting and location**	6	Provide relevant contextual information that may influence findings.	Introduction, Paragraph 2, Page 4; Methods, Economic Modelling, Paragraph 1, Page 4
**Comparators**	7	Describe the interventions or strategies being compared and why chosen.	Introduction, Paragraph 2, Page 4
**Perspective**	8	State the perspective(s) adopted by the study and why chosen.	Introduction, Paragraph 2, Page 4
**Time horizon**	9	State the time horizon for the study and why appropriate.	Methods, Transition Probabilities, Paragraph 4, Page 8
**Discount rate**	10	Report the discount rate(s) and reason chosen.	Methods, Economic Modelling, Paragraph 1, Page 4

For peer review only - http://bmjopen.bmj.com/site/about/guidelines.xhtml

Topic	No.	Item	Location where item is reported
**Selection of outcomes**	11	Describe what outcomes were used as the measure(s) of benefit(s) and harm(s).	Methods, Utility, Paragraph 1, Page 6
**Measurement of outcomes**	12	Describe how outcomes used to capture benefit(s) and harm(s) were measured.	Methods, Utility, Paragraph 1, Page 6
**Valuation of outcomes**	13	Describe the population and methods used to measure and value outcomes.	Methods, Utility, Paragraph 1, Page 6
**Measurement and valuation of resources and costs**	14	Describe how costs were valued.	Methods, Costs, Page 5
**Currency, price date, and conversion**	15	Report the dates of the estimated resource quantities and unit costs, plus the currency and year of conversion.	Methods, Costs, Page 5
**Rationale and description of model**	16	If modelling is used, describe in detail and why used. Report if the model is publicly available and where it can be accessed.	Methods: Economic Modelling, Paragraph 1, Page 4; Transition Probabilities, Paragraph 3, Page 8
**Analytics and assumptions**	17	Describe any methods for analysing or statistically transforming data, any extrapolation methods, and approaches for validating any model used.	Methods: Economic Modelling, Page 4; Transition Probabilities, Page 7; Univariate Sensitivity Analyses, Page 9; and Results, Page 9
**Characterising heterogeneity**	18	Describe any methods used for estimating how the results of the study vary for subgroups.	Not applicable
**Characterising distributional effects**	19	Describe how impacts are distributed across different individuals or adjustments made to reflect priority populations.	Methods: Transition Probabilities, Page 7; and Univariate Sensitivity Analyses, Page 9
**Characterising uncertainty**	20	Describe methods to characterise any sources of uncertainty in the analysis.	Methods, Economic Modelling, Page 4; Transition Probabilities, Page 7; and Univariate Sensitivity Analyses, Page 9
**Approach to engagement with patients and others affected by the study**	21	Describe any approaches to engage patients or service recipients, the general public, communities, or stakeholders (such as clinicians or payers) in the design of the study.	Not reported

Topic	No.	Item	Location where item is reported
**Results**			
**Study parameters**	22	Report all analytic inputs (such as values, ranges, references) including uncertainty or distributional assumptions.	Methods: Costs, Tables 1 – 3, Page 5; Transition Probabilities, Supplementary Tables 1, 2, 3 and 4.
**Summary of main results**	23	Report the mean values for the main categories of costs and outcomes of interest and summarise them in the most appropriate overall measure.	Results, Paragraph 1, Page 9
**Effect of uncertainty**	24	Describe how uncertainty about analytic judgments, inputs, or projections affect findings. Report the effect of choice of discount rate and time horizon, if applicable.	Results , Univariate Sensitivity Analyses, Page 9 and Discussion, Paragraphs 1 and 3, Page 10
**Effect of engagement with patients and others affected by the study**	25	Report on any difference patient/service recipient, general public, community, or stakeholder involvement made to the approach or findings of the study	Not reported
**Discussion**			
**Study findings, limitations, generalisability, and current knowledge**	26	Report key findings, limitations, ethical or equity considerations not captured, and how these could affect patients, policy, or practice.	Discussion, Page 10
**Other relevant information**			
**Source of funding**	27	Describe how the study was funded and any role of the funder in the identification, design, conduct, and reporting of the analysis	End of manuscript, Page 11
**Conflicts of interest**	28	Report authors conflicts of interest according to journal or International Committee of Medical Journal Editors requirements.	End of manuscript, Page 11

From: Husereau D, Drummond M, Augustovski F, et al. Consolidated Health Economic Evaluation Reporting Standards 2022 (CHEERS 2022) Explanation and Elaboration: A Report of the ISPOR CHEERS II Good Practices Task Force. Value Health 2022;25. doi:10.1016/j.jval.2021.10.008
